# Transient misfolding dominates multidomain protein folding

**DOI:** 10.1038/ncomms9861

**Published:** 2015-11-17

**Authors:** Alessandro Borgia, Katherine R. Kemplen, Madeleine B. Borgia, Andrea Soranno, Sarah Shammas, Bengt Wunderlich, Daniel Nettels, Robert B. Best, Jane Clarke, Benjamin Schuler

**Affiliations:** 1 Department of Biochemistry, University of Zurich, Winterthurerstrasse 190, 8057 Zurich, Switzerland; 2 Department of Chemistry, University of Cambridge, Lensfield Road, Cambridge CB2 1EW, UK; 3 Laboratory of Chemical Physics, National Institute of Diabetes and Digestive and Kidney Diseases, National Institutes of Health, Bethesda, Maryland 20892-0520, USA

## Abstract

Neighbouring domains of multidomain proteins with homologous tandem repeats have divergent sequences, probably as a result of evolutionary pressure to avoid misfolding and aggregation, particularly at the high cellular protein concentrations. Here we combine microfluidic-mixing single-molecule kinetics, ensemble experiments and molecular simulations to investigate how misfolding between the immunoglobulin-like domains of titin is prevented. Surprisingly, we find that during refolding of tandem repeats, independent of sequence identity, more than half of all molecules transiently form a wide range of misfolded conformations. Simulations suggest that a large fraction of these misfolds resemble an intramolecular amyloid-like state reported in computational studies. However, for naturally occurring neighbours with low sequence identity, these transient misfolds disappear much more rapidly than for identical neighbours. We thus propose that evolutionary sequence divergence between domains is required to suppress the population of long-lived, potentially harmful misfolded states, whereas large populations of transient misfolded states appear to be tolerated.

## Abstract (web summary)

Single molecule kinetics investigations and molecular simulations are useful tools in elucidating protein assembly mechanisms. Here, the authors use these to show that even naturally occurring tandem repeats undergo transient misfolding and that assembly is much more complex than we previously understood.

## Graphical abstract

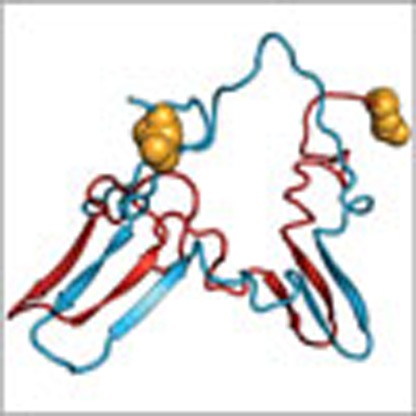

Proteins comprised of a series of covalently linked domains, often sharing a common fold and similar sequence, are highly abundant, particularly in the eukaryotic proteome^1^. The individual domains are usually stable in isolation and largely capable of folding independently, because the energy landscapes of single-domain proteins have evolved to minimize energetic frustration or trapping in alternative conformations stabilized by competing non-native interactions^2^,^3^,^4^. However, in multidomain proteins where neighbouring domains have similar structure, it may be hard to avoid frustration completely, because interactions that stabilize the native fold could also stabilize interdomain misfolded states^5^,^6^,^7^, as demonstrated by the prevalence of domain swapping in the protein universe^8^,^9^,^10^,^11^,^12^,^13^,^14^,^15^,^16^,^17^,^18^.

All-beta, immunoglobulin (Ig)-like domains are involved in many neurodegenerative and non-neuropathic systemic human diseases associated with the formation of deposits or inclusions with amyloid-like characteristics^19^,^20^,^21^,^22^,^23^,^24^. These domains are often found arranged in linear arrays in eukaryotic multidomain proteins, such as the giant human muscle protein titin^25^. Our single-molecule Förster resonance energy transfer (FRET) studies of covalently linked tandem Ig-like domains (Fig. 1a) from the I-band of titin^26^ (I27, I28 and I32) had revealed the formation of a long-lived, domain-swapped misfolded species on refolding^27^ (Fig. 1b; Supplementary Discussion; Supplementary Fig. 1a,b). However, such stable misfolding was found to occur only between domains with sufficiently high sequence similarity^1^,^27^ (Supplementary Fig. 1c), indicating that specific, native-like interactions are responsible for their stability.

Here we combine ensemble kinetics and single-molecule microfluidic-mixing methods with molecular simulations to investigate how sequence divergence affects the interplay of native and non-native interactions in avoiding misfolding and aggregation in tandem Ig-like repeats of these domains^1^,^27^,^28^. In contrast to the apparent simplicity of the previous results close to equilibrium, we find that an unexpectedly wide variety of misfolded states form transiently and to a large extent on the sub-second timescale, even between naturally neighbouring domains. We thus hypothesize that the main function of sequence divergence is not to avoid the occurrence of non-native interactions, but to prevent stable misfolded structures from accumulating.

## Results

### Ensemble kinetics and the effect of non-native interactions

Tandem repeats containing two, three or eight domains of I27 were unfolded in the chemical denaturant guanidinium chloride (GdmCl) and then rapidly diluted to monitor ensemble folding kinetics. Compared with isolated I27 domains, an extra folding phase was observed at low denaturant concentrations (Fig. 2a; Supplementary Fig. 2a). This new, faster phase has the same rate coefficients for all tandem proteins (Fig. 2a), and its relative amplitude increases with the number of repeats, as would be expected for interdomain interactions (Fig. 2b; Methods). Moreover, the native folding phase exhibits a distinct ‘rollover' at low GdmCl concentrations, which is independent of protein concentration (Supplementary Fig. 2d–f) and becomes more pronounced with increasing number of tandem repeats (Fig. 2a): we infer that rapid accumulation of transient misfolded states slows native folding. These results suggest that domain-swapped misfolding can be an even greater problem for multidomain proteins with a large number of repeats.

The considerable amplitudes of the fast refolding phase hint at a surprisingly large misfolded population. For the two-domain construct I27–I27, the amplitude is 23 (±1)%; for the three- and eight-domain constructs, a simple calculation based solely on the number of covalently linked domains predicts that the proportion of misfolded domains would increase by a factor of 1.3 and 1.8, respectively (see the Methods section for details), relative to the two-domain tandem, in good agreement with the measured amplitude increase of 29 (±1)% and 38 (±1)%, respectively (Fig. 2b).

Misfolded species are also detected in ensemble unfolding experiments. Tandem repeat proteins that have been unfolded, refolded (for 2 min) and then unfolded again, reveal the presence of a population of misfolded proteins that unfold faster than correctly folded molecules (Fig. 2a) and are not present in the unfolding of molecules that are unfolded for the first time (Supplementary Fig. 2b,c); again, the fraction of misfolded proteins increases with the number of repeats (Fig. 2a,c), but the rate coefficients of this faster unfolding phase does not vary, suggesting that it originates from the unfolding of misfolded conformations. This conclusion is supported by the agreement of the fast relaxation rate coefficients with the unfolding rate coefficients of the previously identified misfolded subpopulation in single-molecule FRET experiments^27^ (Fig. 2a), and the close match between their relative amplitudes in ensemble experiments (5 (±1)%) and their population fraction (5.0 (±0.2)%) in single-molecule FRET experiments (Fig. 2c).

### Single-molecule FRET kinetics reveal misfolded conformations

**Figure 1:**
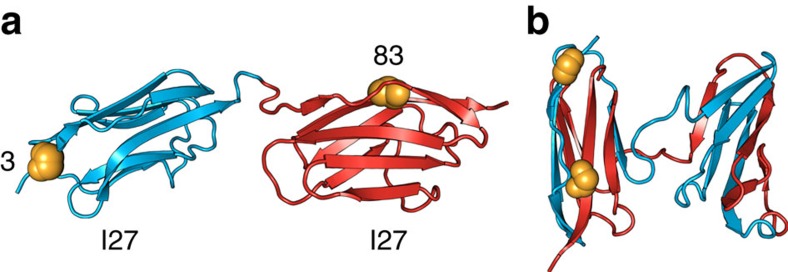
Representative structures of stable alternative conformations of the two-domain tandem construct I27–I27 formed on refolding. (**a**) Native state structure (based on ref. 52). (**b**) One of the possible structures of the strand-swapped long-lived misfolded state formed after refolding by dilution from high GdmCl concentration (based on Gō-like simulations)^27^. Residues 3 of the first domain (blue) and 83 of the second domain (red) are labelled with fluorescent dyes and indicated as orange spheres.

To elucidate the structural basis of the misfolding mechanism and resolve conformational heterogeneity, we used single-molecule FRET. Molecules with different structures will result in different distances between the FRET dyes (Supplementary Fig. 3). Monitoring the efficiencies of energy transfer, *E*, thus enables us to identify the presence of the different species involved. For these investigations, we used two tandem constructs, I27–I27 and I27–I28, both labelled at position 3 of the first domain (A strand) and at position 83 of the second domain (G strand), with Alexa 488 and Alexa 594 as FRET donor and acceptor, respectively (Fig. 1a). Previous single-molecule refolding experiments with I27–I27 had established the formation of a high transfer efficiency misfolded structure, consisting of two strand-swapped I27 domains (Fig. 1b; Supplementary Figs 1b and 3e), which formed within seconds and was stable for days; no indications for misfolding had been found for I27–I28 (ref. 27). To be able to time-resolve the refolding reaction of doubly labelled tandem constructs I27–I27 and I27–I28 (see Methods), we employed a microfluidic-mixing device optimized for single-molecule detection from milliseconds to minutes^29^,^30^. To reach final solution conditions as native as possible and thus maximize the population change, we mixed protein unfolded in 4.5 M GdmCl with buffer at a 1:20 dilution ratio, thereby observing refolding in 0.23 M GdmCl.

Remarkably, the transfer efficiency histograms for I27–I27 (Fig. 3a; Supplementary Fig. 4) show that after 4 ms, we observe not only the expected peak of the fully unfolded state, U^27^–U^27^ (*E*≈0.29), but also a broad range of additional populations with *E* between ∼0.2 and ∼0.9. These persist to ∼1 s, when a simpler picture is regained and the two species detected in previous single-molecule experiments are recovered: the natively folded F^27^–F^27^, and the stably domain-swapped misfolded state, M^27^–M^27^, with mean transfer efficiencies at 0.38±0.02 and 0.90±0.03, respectively^27^ (Fig. 3a; Supplementary Fig. 1b). What is the origin of the broad distribution of transient conformations?

Even without considering misfolding, other populations are expected to contribute at early refolding times: molecules with both domains unfolded (U^27^–U^27^; Supplementary Fig. 3a) and with only one domain folded, (F^27^–U^27^/U^27^–F^27^; Supplementary Fig. 3b). To obtain the transfer efficiency of the completely unfolded state in 0.23 M GdmCl, we measured U^27^–U^27^ and U^27^–U^28^ between 4.6 and 0.5 M GdmCl ∼4 ms after mixing (when the totally unfolded population is >95%) and extrapolated their transfer efficiency to 0.23 M GdmCl (Fig. 4a,b), yielding *E*=0.27±0.02 and *E*=0.32±0.03, respectively. A similar procedure was employed to identify the transfer efficiency of F^27^–U^27^ and F^27^–U^28^. In this case, we took advantage of the slow refolding of I28 compared with I27 (∼4 × 10^−3^ s^−1^ versus ∼15 s^−1^, respectively, in 0.23 M GdmCl (refs 31, 32)), allowing us to transiently populate F^27^–U^28^ in the I27–I28 tandem and obtain its transfer efficiency of 0.28±0.02 in 0.23 M GdmCl (Fig. 4c). In view of the positions of the fluorophores in the tandem repeats, both F^27^–U^27^ and U^27^–F^27^ are expected to exhibit this transfer efficiency, within experimental uncertainty.

### Role of native-like interactions in misfolding

The stable misfolded domains M^27^–M^27^ consist of two strand-swapped domains, each similar in structure to a native I27 (M3 in Fig. 5). One of these domains forms via misfolding of the central segments of the I27–I27 polypeptide chain to form a ‘central domain' (CD), which resembles a circular permutant of I27 (see, for example, topological diagrams in Supplementary Fig. 5, where it is clear how strand swapping results in a rewiring of the (mis)folded state, such that the strands F and G are no longer the termini of the protein), while the other ‘terminal domain' (TD) is composed of the remaining segments closer to the chain termini. On the basis of the sequence separation of contacts in each of these structures, it is plausible to assume that the entropy cost for forming interactions between β-strands recruited from the central part of the unfolded I27–I27 polypeptide chain is much lower than for the same process to occur between β-strands at the opposite ends of the chain (Supplementary Fig. 3d). Consequently, it seems likely that a misfolded structure with the central domain (mis)folded but the terminal domain still unfolded is a metastable refolding intermediate (
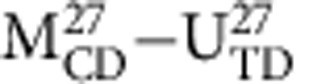
 or M1 in Fig. 5 and Supplementary Fig. 3d). Using Gō-like model simulations similar to those in our previous work^27^, we obtained six possible strand-swapped species, differing in the identity and number of strands exchanged on refolding of I27–I27. This observation suggests that six different central domains can be formed during refolding *en route* to the high transfer efficiency, strand-swapped state: indeed, such species occur as intermediates in the simulations.

To test whether misfolded structures with the central domain (mis)folded but the terminal domain still unfolded are stable enough to be metastable refolding intermediates, we engineered and produced the circular permutants corresponding to the central domains of misfolded species as seen in the simulations. Equilibrium denaturation experiments showed that these proteins were strongly destabilized compared with the wild-type structure, but most of them were at least partially folded under native conditions (Supplementary Fig. 5a). An analogous simulation model for the circular permutants derived from the Gō-type model used to describe domain swapping, showed that the permutants were roughly fourfold less stable than wild-type I27, a destabilization similar to that seen in experiment (Supplementary Fig. 5b). Taken together, these observations strongly suggest that analogous misfolded structures (
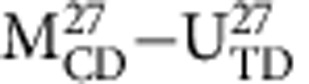
) will be populated at least transiently during refolding until stabilized by the formation of the misfolded terminal domain.

Simulations suggest that, given the positions of the fluorescent dyes on the N- and C-terminal strands of the first and second domain, respectively, all putative intermediates with the central domain (mis)folded are expected to yield transfer efficiencies similar to that of one unfolded I27 monomer labelled at equivalent positions, because folding of the central domain effectively shortens the chain separation of the labels by the length of one I27 domain (see structural representations of 
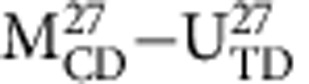
, in Fig. 5 and Supplementary Fig. 3d). To assign the transfer efficiencies of these central domain misfolds, we thus measured the transfer efficiency of a labelled, unfolded I27 monomer at low GdmCl concentration by populating it transiently in the microfluidic mixer (Fig. 4d). The resulting value of *E*=0.49±0.03 lies in the previously unassigned region of the FRET efficiency histogram populated at early times during I27–I27 refolding, suggesting that 
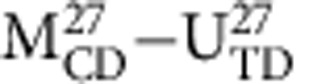
 (M1) represents at least part of the additional species observed.

Note that we cannot exclude a misfolded state with the terminal domain folded and the central domain unfolded (
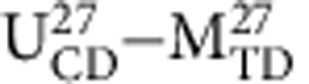
) as an alternative intermediate *en route* to the fully misfolded state M^27^–M^27^ (or M3 in Fig. 5). However, formation of the terminal domain before the central domain would be indistinguishable from M^27^–M^27^, because the dyes are on the terminal segments of the sequence and will be locked in their position on terminal domain (mis)folding, such that the subsequent folding of the central domain will not cause a FRET efficiency change. Occurrence of this 
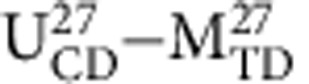
 species would, therefore, have the effect of accelerating the appearance of M^27^–M^27^ to a degree dependent on its abundance, which is expected to be small because of the high entropic cost associated with the terminal domain (mis)folding first. Given that M^27^–M^27^ is *per se* a small population, we would not be able to detect such an effect and thus did not include this pathway in the model used to fit the data.

However, even after including 
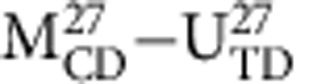
 and all intermediate species that can plausibly be formed based on native and native-like interactions, the transfer efficiency range around 0.65 is still unaccounted for, in particular between ∼10 and 40 ms of I27–I27 refolding, prompting us to consider additional types of misfolded states.

### Intramolecular amyloid-like misfolded states

Computational investigations of tandem repeats of I27 by Wolynes and co-workers^33^ predicted an alternative class of misfolded structures stabilized by interactions between parallel β-strands, analogous to those found in β-amyloid. To test whether such a model could be consistent with the observed single-molecule kinetics, we extended our earlier folding simulations by allowing the possibility for such structures to form, using an empirical energy function based on amino-acid-specific interactions that stabilize β-sheet hydrogen bond patterns in native structures of globular proteins^34^ (see Methods and Fig. 5 for representative examples, denoted M2). Starting multiple folding runs from an initial extended state, we indeed obtain species characterized by formation of *i,i+L* self-interactions between a residue and the corresponding residue in the adjacent domain (*L*=93, the length of one I27 and linker). They are characterized by the parallel pairing of the same strand from two domains, and most of them include the self-recognizing region 56–61 previously identified^33^. By averaging over many folding simulations, we can reproduce both the rapid initial formation of such misfolded species, which resemble an ‘intramolecular amyloid', and their eventual unfolding and conversion to either natively folded or domain-swapped species (Fig. 3e).

### A unified kinetic scheme for folding and misfolding

On the basis of the results presented above and our extensive knowledge of I27 stability and folding kinetics and mechanism^31^,^32^,^35^,^36^, we are now able to combine all the information at our disposal to model the time-dependent FRET histograms that we collected in the microfluidic mixer with the simplest physically plausible mechanism. Extensive additional information is available that aids the choice of a kinetic mechanism and markedly reduces the number of free fit parameters. Apart from the unfolded state U^27^–U^27^, whose transfer efficiency we obtained from independent measurements (*E*=0.27±0.02; Fig. 4a), we also know the transfer efficiency of the two species formed along the productive folding pathway, F^27^–U^27^/U^27^–F^27^ (*E*=0.27±0.02) and F^27^–F^27^ (*E*=0.38±0.02), from independent measurements in the microfluidic mixer (Fig. 4c) or from equilibrium measurements of never unfolded (or refolded) I27–I27 (Supplementary Fig. 1a), respectively. In addition, we know the folding rate coefficients for the folding of native I27 and I28 at the relevant GdmCl concentration from ensemble stopped-flow experiments^31^,^32^. We also have an independently derived value for the transfer efficiency of the species with the central domain (mis)folded and the terminal domain still unfolded, 
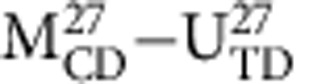
 (*E*=0.50±0.02; Fig. 4d), which is likely to be the main species populated *en route* to the fully misfolded, long-lived state M^27^–M^27^ because the entropy cost for forming interactions between β-strands recruited from the central part of the unfolded I27–I27 polypeptide chain is much less than for the same process to occur between β-strands at the opposite ends of the chain (Supplementary Fig. 3d). Moreover, we have measured the transfer efficiency of M^27^–M^27^ in equilibrium refolding experiments^27^ (Supplementary Fig. 1b), and we know the rate coefficient for its back-conversion to the native state in 0.23 M GdmCl (∼10^−5^ s^−1^)^27^. Thus, there are already five species that need to be populated during I27–I27 refolding, and which we thus have to include in a realistic kinetic model, and two rate coefficients that will inform our fit further. Crucially, we also have estimates of the corresponding peak shapes from the FRET efficiency histograms as a function of GdmCl concentration (Fig. 4; Supplementary Table 1).

This wealth of prior knowledge places essential constraints on the minimum number of species that need to be included in the model, their kinetic connectivity, some of the rate coefficients and the shapes of the transfer efficiency peaks. The robustness of our analysis (verified by parameter randomization; Supplementary Fig. 6) results from the combination of these constraints with a global fit of the entire transfer efficiency time series that requires the kinetic model to yield a satisfactory fit of the data at all times, both at stages where only two populations would seem sufficient for a good fit (for example, after 2 s) and when the largest number of species is apparent (for example, around 40 ms). Using this procedure, in addition to the five species listed above, at least one population with a broad transfer efficiency distribution was required for fitting the transfer efficiency histograms at *E*≈0.65, which corresponds to the proposed amyloid-like population M2. For comparison, we show in Supplementary Fig. 7 fits of the data without a sixth species, either without the amyloid-like population M2 or without the strand-swapped misfolded state M1 (
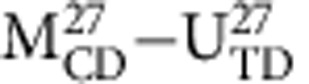
), to illustrate that a minimum of six species is required for describing the transfer efficiency histogram time series.

We want to emphasize, however, that our kinetic model is the simplest description, both structurally and kinetically, that is able to describe the data while satisfying all independent constraints; we cannot exclude additional species with strongly overlapping transfer efficiency distributions to be transiently formed during refolding. The model also successfully reconciles single-molecule and ensemble refolding data: The fast phase in the ensemble refolding data corresponds to the rapid accumulation of both native-like (M1 and M3) and amyloid-like (M2) misfolds, as well as the direct formation of the native state from unfolded; the native refolding phase is slowed by the need to escape from the misfolded states.

### Relevance of transient misfolding for natural titin sequences

**Figure 2:**
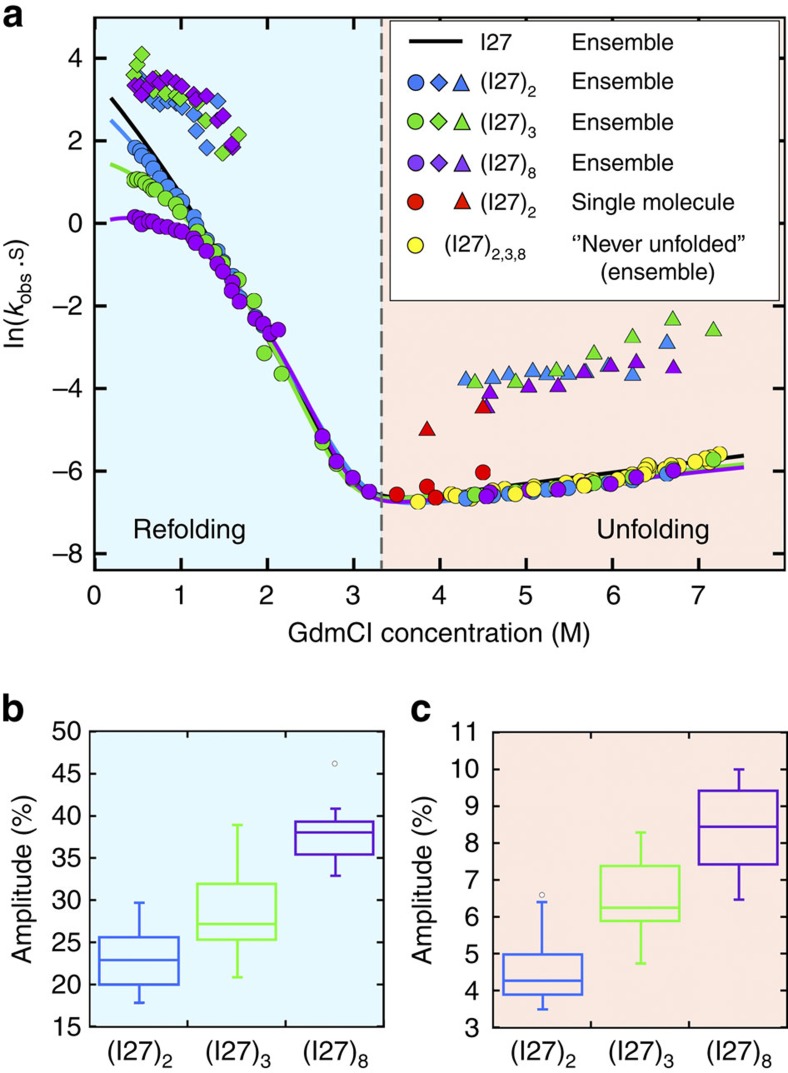
Ensemble kinetics of I27 tandem constructs. (**a**) Ensemble refolding and unfolding kinetics of I27 single-domain (black), two- (blue), three- (green) and eight-domain (purple) constructs. All multidomain constructs display two refolding phases (circles and diamonds), with a deviation from linearity at low GdmCl concentration (rollover) and two unfolding phases (circles and triangles). Unfolding of all ‘never unfolded' I27 tandem constructs is well described by a single-phase corresponding to native-state unfolding (yellow circles). Rate coefficients for both slow and fast unfolding phases match values for the native (red circles) and the stable misfolded state (red triangles) unfolding measured in single-molecule experiments^27^, respectively. (**b**,**c**) Comparison of the relative amplitudes for the misfolding phase of all constructs observed during refolding (**b**) and unfolding (**c**) of previously refolded protein. For both reactions, the proportion of misfolded species increases with the number of domains in a manner we would predict (1:1.3:1.8, equation 1 in Methods).

**Figure 3:**
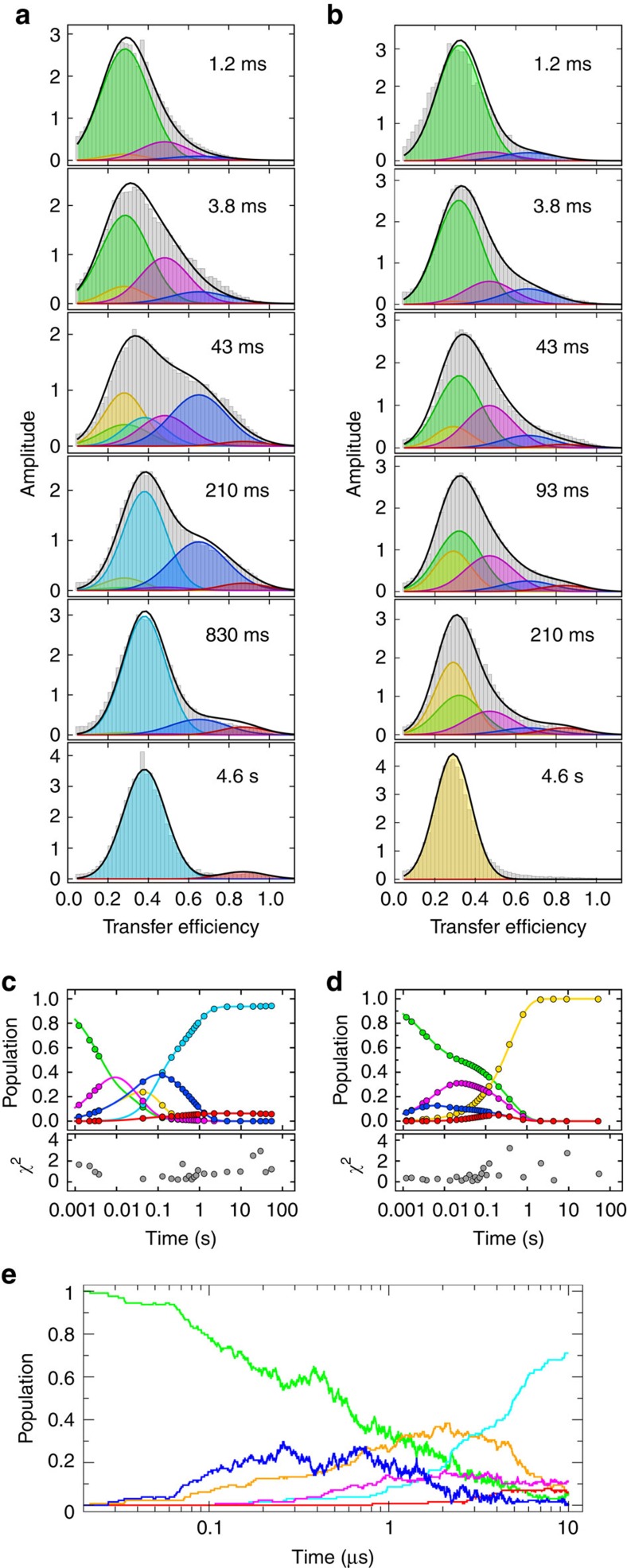
Single-molecule kinetics of doubly labelled I27–I27 and I27–I28 refolding. (**a**,**b**) A selection of I27–I27 (**a**) and I27–I28 (**b**) transfer efficiency histograms (grey) recorded at increasing times (insets) after initiation of refolding in 0.23 M GdmCl in the microfluidic mixer. Gaussian fits of individual populations to the global kinetic model (Fig. 5) are colour coded as follows: green, fully unfolded (UU); yellow, one domain folded, one unfolded (FU/UF); cyan, natively folded (FF); magenta, strand-swapped misfolds with central domain formed (M1); red, strand-swapped fully misfolded species (M3); blue, non-native-like misfolds (M2; see main text); nomenclature as in Fig. 5. The sums of all populations are shown as black lines. Every histogram shown is the average of two or more independent measurements. (**c**,**d**) Plots of the time evolution of each population of I27–I27 (**c**) and I27–I28 (**d**) according to the rate coefficients obtained from the global fit of the entire histograms time series (Supplementary Figs 4 and 8a); circles indicate times where a histogram was measured. Panels below show the sum of the squared residuals (*χ*^2^) for every histogram (equation 8 in Methods). (**e**) Coarse-grained folding simulations displaying the relative population of each state (colour-coded as above), including the intramolecular amyloid-like misfolds M2 (blue), as a function of time.

What is the relevance of these findings for naturally occurring multidomain proteins? The investigation of tandem repeats of the natural neighbours I27 and I28 allows us to answer this question. Contrary to I27–I27, which forms the stably misfolded state at *E*≈0.9 (Fig. 2a), tandem repeats of domains with low sequence similarity, such as the natural neighbours I27 and I28 (24% sequence identity), show no stable misfolding^27^. To our surprise, in single-molecule microfluidic-mixing experiments with I27–I28 labelled in the same way as I27–I27, we observed similarly broad transfer efficiency distributions (Fig. 3b) as for I27–I27 on the sub-second timescale, including, most strikingly, a small population with the same transfer efficiency as the stable misfolded state of I27–I27 at *E*≈0.9 (Fig. 3b; Supplementary Fig. 8a). However, all of these states are only populated transiently and fall below detectable levels after about 0.5 s, that is, much faster than in I27–I27 (Fig. 3a,b; Supplementary Table 1). Note that the fully native state cannot be attained within the time window of the microfluidic-mixing experiment because of the slower refolding of I28 (ref 31); as a result, the FRET efficiency histograms in the seconds range are dominated by F^27^–U^28^ at *E*≈0.28. Remarkably, the same minimum number of populations is required to describe the early histograms of I27–I28 as for I27–I27, including the amyloid-like species at *E*≈0.65, only with the exception of fully folded I27–I28, which forms on a much longer timescale (4 × 10^−3^ s^−1^; Fig. 3b; Supplementary Fig. 8a).

## Discussion

Our investigation of the refolding kinetics of I27–I27 and I27–I28 reveals heterogeneous misfolding, whose unexpected extent and complexity is largely independent of interdomain sequence identity, leading to the appearance of a surprisingly broad range of populations in the single-molecule FRET experiments (Fig. 3) and an additional fast phase and rollover in the ensemble refolding kinetics (Fig. 2a). Molecular simulations suggest that the largest misfolded populations observed in experiment (M2) are not native-like domain-swapped structures but non-native-like, interdomain misfolds (M2) characterized by self-complementary interactions between each residue and its twin in the adjacent domain, due to the high amyloid propensity of parts of the I27 sequence^33^. These species have been proposed to act as seeds for the aggregation of repeats of Ig-like domains^37^ observed at high protein concentrations^28^. The appearance of such misfolds even for the tandem of natural neighbours, I27–I28, implies that transiently stable non-native-like structures can also be formed with non-identical sequences. This conclusion is supported by the similarity of a knowledge-based score for parallel-β interactions between I27 and I28, and between two identical I27 domains^34^ (Supplementary Fig. 9), suggesting that this misfolding mechanism could be widespread for proteins rich in β-structure.

The remainder of the misfolded populations is represented by topologically native-like species (M1) comprising structures with a misfolded central domain and an unfolded terminal domain, transiently accumulated in both I27–I27 and I27–I28 *en route* to the stable misfolded conformation (M3 with *E*≈0.9). The presence of such high transfer efficiency species during I27–I28 refolding was most unexpected, and suggests that, contrary to our previous conclusions and expectations, the natural neighbours I27 and I28 can actually form strand-swapped structures, M^27^–M^28^, analogous to those seen in I27–I27, albeit far less stable. The ability to form such structures based on native-like interactions in Ig-like multidomain proteins may be connected to the folding mechanism of the individual domains, and may thus affect other protein families^38^,^39^ with a topology conducive to swapping of secondary structure elements^40^. The 
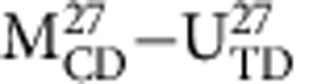
 misfolded states resemble folded domains and are thus likely to require the recruitment of the same conserved set of residues constituting the folding nucleus to form^35^,^36^,^41^,^42^. Such residues in I28 are probably similar enough to those of I27 to enable the formation of these native-like misfolded structures: although the overall sequence identity between I27 and I28 is only 24%, we note that 40% of the buried residues most important for the transition state for folding^32^ (with Φ-values >0.5) are identical and a further 33% are highly similar. However, the thermodynamic stability of such native-like misfolded states will still be determined by the size and strength of the whole interaction network, which depends on the overall sequence identity of the swapping partners, and is probably too low for these species to be long lived.

The picture emerging from our results is that avoiding misfolding in multidomain proteins due to the high local concentration of similar protein segments may pose evolutionary challenges analogous to those of avoiding intermolecular aggregation^43^ at the high cellular protein concentrations^44^. In particular, for all-β proteins, such as the Ig-like domains, the high local concentration of β-strands in the denatured state and the correspondingly large number of potential hydrogen bonding partners could lead to the rapid, entropically favoured formation of a broad range of β-sheet-like structures. However, such misfolded structures involving only two domains are likely to be unstable, accounting for the subsequent unfolding and conversion to the correct, native fold. Indeed, molecular simulations allowing for the formation of native, native-like (M1 and M3), and non-native-like (M2) interactions result in a transient population of both types of intermediates (Fig. 3e), which mostly convert to the correctly folded protein at longer times. Structures of this type could represent a rather general phenomenon, especially in multidomain proteins rich in β-structure.

We have shown that the stable, long-lived misfolded species observed previously^27^ are just one facet of a complex mechanism modulated by protein topology and the properties of the polypeptide sequence. The observation of transient misfolded species even for the naturally occurring tandem repeat I27–I28 demonstrates how finely the propensities of folding and misfolding must be balanced by coevolution of adjacent domains to avoid the formation of stable misfolded states, and strongly suggests that the sequence divergence of neighbouring domains serves to destabilize potential misfolded structures, rendering them short lived and thus minimizing their detrimental impact on multidomain protein function and, perhaps even more importantly, avoiding aggregation^19^,^20^,^37^,^43^,^45^,^46^.

## Methods

### Circular permutants

In the predictions from Gō-like simulations, the central domain always comprised residues from the C-terminal portion of domain 1 and the N-terminal portion of domain 2, thus it resembles a circular permutant of I27 (ref 27). All possible strand-swap permutants were designed with a linker (–RSEL–) that is present in any domain-swapped misfolded species between the original N and C termini. All synthetic genes were provided by GenScript (USA) in a pUC-holding vector and were ligated into the expression vector, a modified version of pRSETa.

### Ensemble kinetics experiments

*Refolding*. Unfolded protein in 5 M GdmCl underwent rapid mixing 1:10 (v/v) with PBS (10 mM sodium phosphate (pH 7.4), 137 mM NaCl, 2.7 mM KCl) and the refolding kinetics were recorded using an AppliedPhotophysics SX20 stopped-flow fluorimeter.

*Unfolding*. The kinetics of unfolding were recorded following manual mixing (1:10) on a PerkinElmer fluorimeter at final concentrations of 4–8 M GdmCl.

*Unfolding of refolded protein*. Unfolded protein in 5 M GdmCl was allowed to fold by a 10 × dilution with PBS. The final reaction solution is referred to as ‘previously refolded'. For practical reasons, the time allowed for refolding was 2 min, the refolding half-life of native I27 is ∼70 ms. Excitation was at 280 nm and emission was followed at or above 320 nm. All experiments were conducted in a final dithiothreitol concentration of 5 mM, at 25 °C.

### Ensemble equilibrium experiments of circular permutants

Given the marginal stability of the circular permutants in native conditions, GdmCl-induced denaturation measurements were conducted in the presence of 1 M glucose. The stabilization due to the addition of glucose was measured for the wild-type protein and this contribution (1.2 kcal mol^−1^), assumed equal for all I27 variants, was then subtracted from the stability of the circular permutants measured in the presence of glucose, thus attaining a reliable estimate of these species' stability in native buffer.

### Predicting the population of misfolded species

The proportion of misfolded domains was predicted for constructs of 1–8 repeat domains by assuming that the probability of domain *i* misfolding is proportional to the number of directly neighbouring domains (*n*_*i*_). The average number of neighbouring domains for each domain within a tandem construct of length *N* is given by


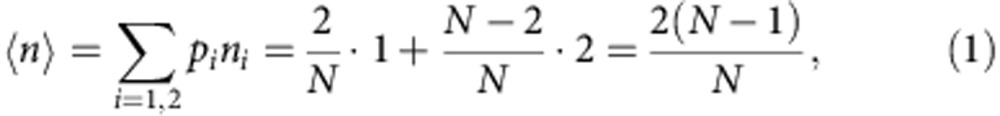


where *p*_*i*_ is the fraction of domains with *n*_*i*_ neighbours. Thus, the ratio of 〈*n*〉 values for constructs of different length, *N*, would be expected to match the relative abundances of misfolds extracted from the amplitudes obtained in kinetics experiments. This approach neglects the possibility of misfolding between non-neighbouring domains and the inhibition of misfolding by already misfolded domains.

### Protein preparation and labelling for single-molecule experiments

Cysteine residues were introduced by site-directed mutagenesis: E3C in domain 1 (always I27) and N83C in domain 2 if I27, and K83C for I28 (with the numbering relative to a single domain). DNA sequencing confirmed the mutagenesis. I27–I27 and I27–I28 tandems, with the engineered surface cysteines, were expressed as described previously^31^,^47^. Labelling was carried out using Alexa Fluor 488 (donor) and Alexa Fluor 594 (acceptor) maleimide (Invitrogen) according to the manufacturer's procedures. Both dyes were mixed simultaneously with reduced protein in equimolar ratios and incubated at 4 °C for ∼10 h. Unreacted dye was removed by gel filtration and the differently labelled variants were separated by ion-exchange chromatography (MonoQ 5/50 GL; GE Healthcare Biosciences AB, Uppsala, Sweden). I27 has two intrinsic cysteines that were not removed as they are buried in the native state and all labelling was carried out on folded protein in native conditions.

### Single-molecule instrumentation

Observations of single-molecule fluorescence were made using a custom-built confocal microscope equipped with a continuous wave 488-nm solid-state laser (FCD488-010, JDSU, Milpitas, CA, USA) and an Olympus UplanApo × 60/1.20 W objective. After a dichroic mirror that separates excitation and emission light (500DCXR, Chroma Technology, Rockingham, VT, USA), fluorescence emission passed through a 100-μm pinhole and was split by a second dichroic mirror (585DCXR, Chroma Technology) into donor and acceptor fluorescence. Donor fluorescence then passed a filter (ET525/50M, Chroma Technology) before being focused onto a single-photon avalanche diode (MPD 100ct, Micro Photon Devices, Bolzano, Italy), while acceptor fluorescence passed a filter (QT 650/100) before being focused onto a single-photon avalanche diode (SPCM-AQR-13, PerkinElmer Optoelectronics, Vaudreuil, QC, Canada). The arrival time of every photon was recorded with a two-channel time-correlated single-photon counting module (PicoHarp300, PicoQuant, Berlin, Germany).

### Single-molecule kinetics measurements

Rapid-mixing experiments were performed with a microfluidic-mixing device fabricated by replica moulding in polydimethylsiloxane (PDMS) described in refs 29, 30. Fully denatured protein in 4.56 M GdmCl, PBS pH 7.4 and 10 mM β-mercaptoethanol (Sigma) entering the microfluidics device from the central inlet channel was diluted 1:20 with either PBS at pH 7.4 or with various concentrations of GdmCl in PBS buffer at the same pH, entering via the two side inlet channels, resulting in final GdmCl concentrations between 4.56 and 0.23 M. The average flow rate was kept at 1 mm s^−1^ in all cases by varying the pressure applied to the inlet channels according to the viscosity of the solutions^30^. The stability of the flow was monitored by analysing the fluorescence intensity cross-correlation of the measurements in the observation channel. Measurements were performed at 22 °C with a protein concentration ranging between 5 and 25 pM after mixing, in PBS pH 7.4, 140 mM β-mercaptoethanol and 0.01% Tween 20 (Pierce) with varying concentrations of GdmCl (Pierce). Tween 20 was used to prevent surface adhesion of the proteins^48^, and the photoprotective agent β-mercaptoethanol was employed to minimize chromophore damage and enhance brightness^49^. Data were collected for a time varying between 15 min and 10 h depending on flow rate, protein concentration and laser power, which varied between 100 and 300 μW, measured at the back aperture of the objective (beam waist: 8mm). Bursts of fluorescence photons were identified by combining photons detected within 150 μs from each other, and events comprising 35 or more photons were used for the subsequent analyses. Transfer efficiencies were corrected for quantum yields, cross talk and direct excitation as described previously^50^. GdmCl concentrations were measured with an Abbe refractometer (Krüss, Germany).

### Analysis of the time-resolved transfer efficiency histograms

Each time-resolved transfer efficiency histogram was measured between two and five times in different experiments on different days and averaged. Only the parts of the transfer efficiency histogram above *E*=0.05 were used and normalized to a total area of one. As a result, we have a time series of *N* histograms (*N*=23 for I27–I27 and *N*=25 for I27–I28) measured at times *t*_*n*_ (*n*=1, ..., *N*) after mixing. Each histogram can be written as a vector


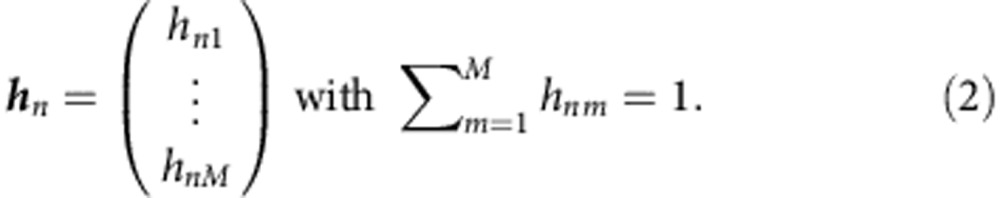


Here *h*_*nm*_ with *m*=1, ..., *M* is the number of burst events with transfer efficiency values *E* satisfying *E*_m_−Δ/2<*E*≤*E*_*m*_, where Δ=0.02 is the bin width, and *E*_*m*_ is the mean transfer efficiency value of the *m*th bin. We fit the histograms globally with


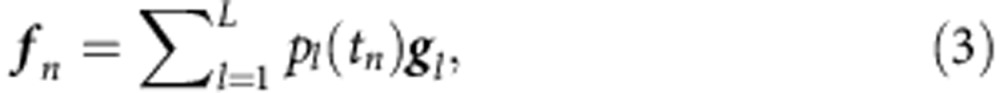


where *p*_*l*_(*t*_*n*_) is the relative population of state *l* at time *t*_*n*_ assuming a total of *L* states (*L*=6 for the kinetic model of Fig. 5). The components of the vectors **g**_l_ are given by a Gaussian peak function:


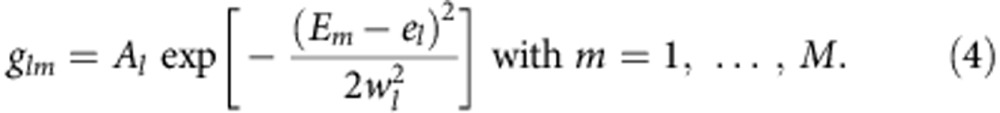


Here *e*_l_ is the peak position, and *w*_*l*_ is the width of the peak describing the species *l*. *A*_*l*_ is a normalization factor that makes 
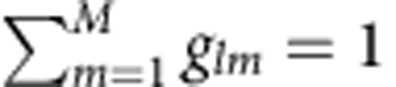
. The time dependence of the relative populations *p*_*l*_(*t*_*n*_) results from our six-species kinetic model (Fig. 5) as:


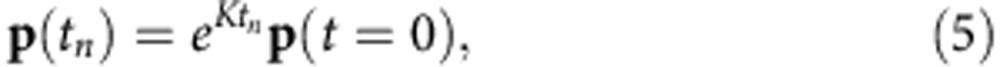


where **p**(*t*_*n*_) is a population vector of the form


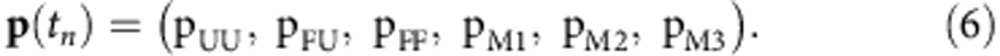


**K** is the matrix of the interconversion rate coefficients that describes the population change of the six species over time (Fig. 5):


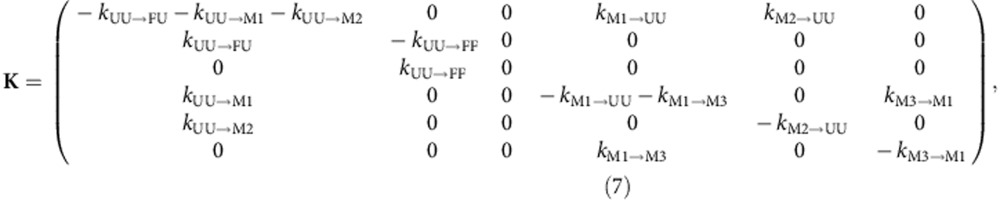


and **p**(0)=(1, 0, 0, 0, 0, 0) is the population vector at time zero, starting from the fully unfolded state. In the global fitting procedure, we minimize


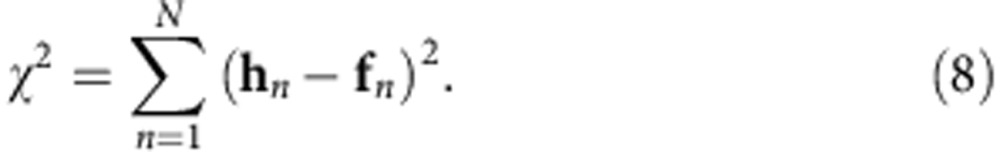


Deviations of the sum of the fitted populations from the measured data were calculated for each bin of every histogram as the residuals:


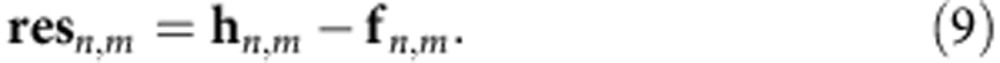


The peak positons and widths *e*_l_ and *w*_l_ , which we assume to be constant over time, were fixed to values obtained either from equilibrium experiments (FF and M3), extrapolated from the dependencies on GdmCl (UU, FU/UF and M1), or resulting from fits of individual histograms with the peak parameters of all other populations constrained (M2). In addition to these values, we also knew the native folding rate coefficient *k*_FU/UF→FF_=15±5 s^−1^ (=0.5 *k*_UU→FU_) and the rate coefficient for M3 unfolding in 0.23 M GdmCl (*k*_M3→M1_∼2 × 10^−5^ s^−1^). Instead of fixing the native folding rate coefficient, we minimized *χ*^2^ under the constraint that its value must be within the given uncertainty interval. Note that k_UU→FU_ is twice the value of k_FU/UF→FF_ because from the fully unfolded state the probability for a folding event to occur is twice as high. All other rate coefficients were left unconstrained. The extremely slow M3 unfolding indicates that k_M3→M1_ is much smaller than k_M1→M3_ and k_M1→UU_, which, together with the small M3 population, implies a large uncertainty associated with the exact value of k_M3→M1_ fitted from the data (experiment measurement time is <1 min). The rate coefficients found by the fit, as well as the corresponding set of peak positions and widths, are reported in Supplementary Table 1, while the fits are depicted in Fig. 3a, b and Supplementary Figs 4, 7 and 8.

### Robustness of the fitted rate coefficients with respect to fixed parameters

To evaluate the sensitivity of the fitted rate coefficients to the fixed input parameters, transfer efficiencies and widths, starting from values chosen as explained above, were varied simultaneously, assuming that they were normally distributed around the original value, with a standard deviation of 5% of that value. Analogously, k_UU→FU_ and k_M3→M1_ were varied randomly assuming that the values were normally distributed with a s.d. of 50% of their original values (30 and 10^−5^ s^−1^, respectively). After each parameter randomization for the fixed positions, widths and rate coefficients, a fit was performed and the outcome recorded, for 10^4^ cycles. Results of this procedure are summarized in Supplementary Fig. 6, where all positions, widths and rate coefficients yielding fits with *χ*^2^ within 50 or 5% of the best *χ*^2^ are histogrammed (see Supplementary Table 1). Distributions of the resulting mean transfer efficiencies, widths and rate coefficients yielding fits with low *χ*^2^ also show clear global maxima very close to the optimal fit values identified, indicating that the fit results are robust.

### Five-species fit of the FRET efficiency histograms for I27–I27 refolding

To demonstrate the need for six species to satisfactorily describe the single-molecule data, we show in Supplementary Fig. 7a,d an attempt to fit the time-resolved histograms with a five-species model excluding either the M2 or M1 populations, respectively. It is evident that these fits do not fully describe the experimental data; the *χ*^2^ values of the five-species fits are ∼131 and 55, respectively, versus ∼22 for the six-population fit.

### Possible role of disulfide bonds to the FRET efficiency distributions

We considered the possibility of intramolecular disulfide bonds as an alternative origin of the broad distributions of populations that we observed in both ensemble and single-molecule experiments. The I27 monomer is 89 residues long, and contains Cys residues at positions 47 and 63, meaning that an intradomain disulfide bond will be far more likely to form than one between two different domains. However, both in previous ensemble kinetics of the individual domains^32^ and in the current time-resolved single-molecule experiments, we found no evidence that disulfide bonding occurs, probably owing to the large concentration of reducing agent used (5–55 mM dithiothreitol and 140 mM β-mercaptoethanol for ensemble and single-molecule experiments, respectively.

### Fits of the transfer efficiency dependencies on GdmCl

**Figure 4:**
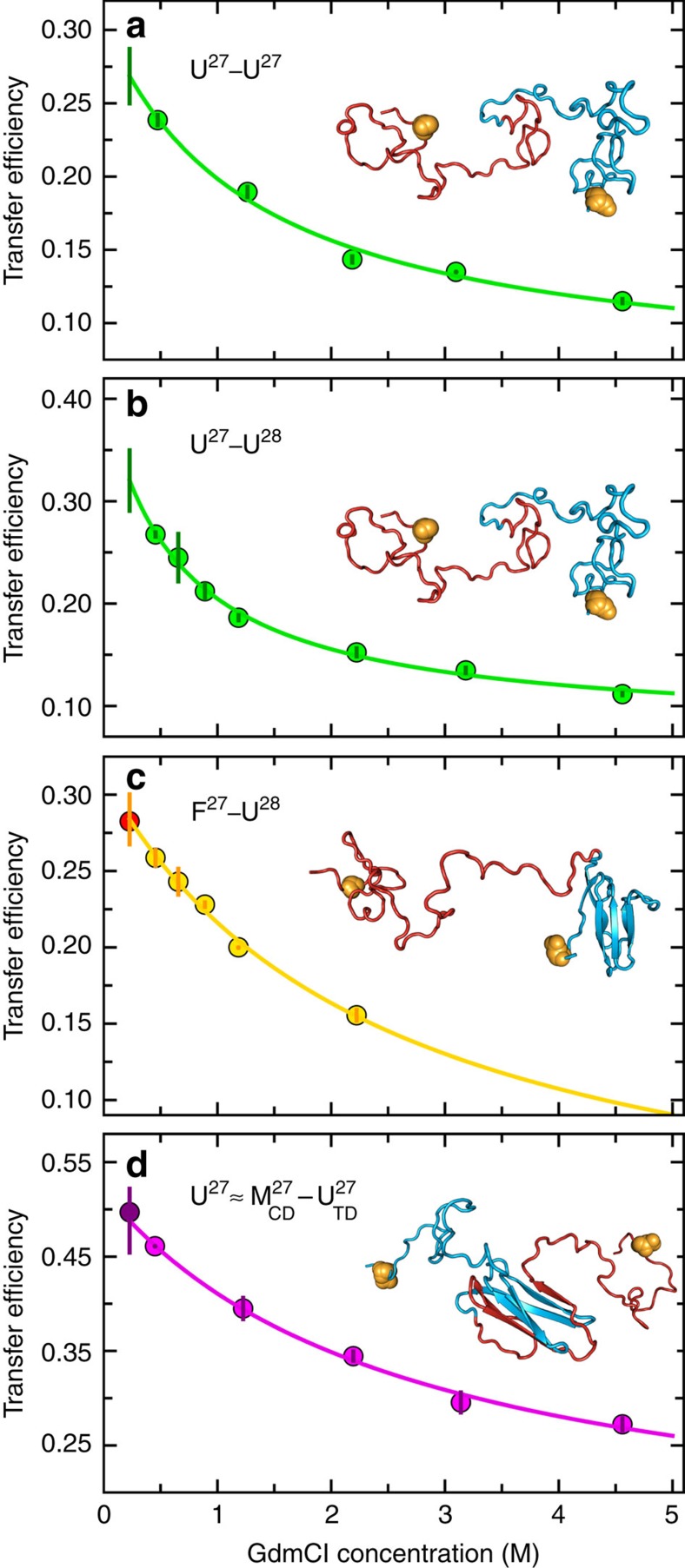
Dependence of the transfer efficiencies of individual species on GdmCl concentration. (**a**,**b**) Transfer efficiency values obtained for the population of unfolded molecules by fitting FRET efficiency histograms recorded in the microfluidic-mixing device at different final GdmCl concentrations 3.8±0.3 ms after mixing. (**c**) Same experiments as in **b**, but histograms here were recorded after 4.6±0.5 s to allow for complete refolding of I27 in the presence of unfolded I28 (see main text). (**d**) I27 monomer transfer efficiencies measured 3.8±0.3 ms after mixing at different concentrations of GdmCl. The transfer efficiency of this species is taken to be representative of a misfolded state with the central domain formed and the terminal domain unfolded (
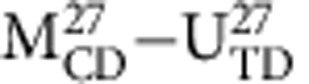
, see main text). In all plots, a solid vertical bar at 0.23 M GdmCl, that is, the GdmCl concentration in which we measured refolding, represents the uncertainties obtained from the 90% confidence interval of an extrapolated fit (equation 10 in Methods) to all data points excluding the one at 0.23 M GdmCl (error bars indicated for individual data points). For the plots in **c** and **d**, it was possible to measure the transfer efficiency directly at 0.23 M GdmCl (red and purple circle, respectively), but these points were not included in the fits: the excellent agreement of the extrapolated and measured transfer efficiency values confirms the suitability of the extrapolation approach. A representative structure of the corresponding molecular species is given in each panel; the residues labelled with fluorescent dyes are indicated as orange spheres. All plots are colour coded according to the transfer efficiency populations in Fig. 3.

**Figure 5:**
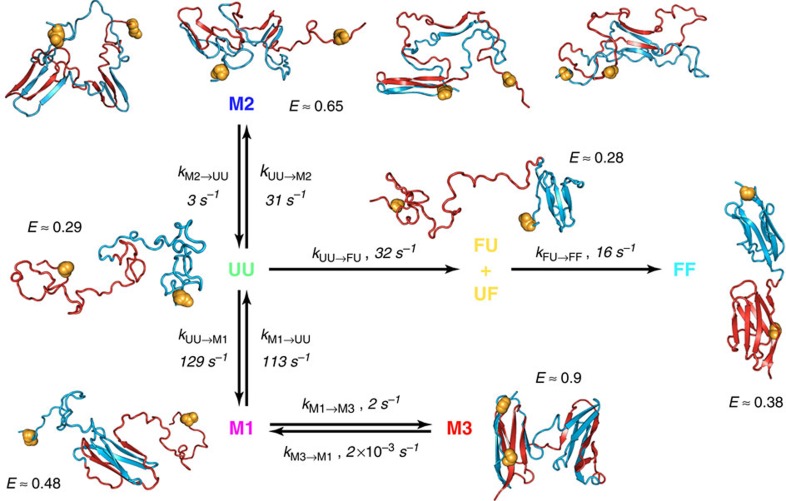
Kinetic scheme for I27–I27 and I27–I28 refolding including representations of the proposed structures. UU is the fully unfolded state (U^27^–U^27^); FU/UF have one domain correctly folded and the other unfolded (F^27^–U^27^ or U^27^–F^27^); FF is the natively folded state (F^27^–F^27^); M3 has both the central and terminal domains misfolded (M^27^–M^27^); M1 is a strand-swapped misfold with the central domain formed and the terminal domain unfolded (
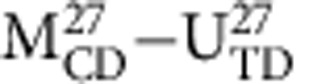
); M2 indicates a collection of misfolded structures named ‘intramolecular amyloids', characterized by non-native pairing of two or more beta strands (see main text). The residues labelled with fluorescent dyes are indicated as orange spheres. Single-molecule data for I27–I27 and I27–I28 refolding (Fig. 3; Supplementary Figs 4 and 8a) are fitted with this model, but in the time window covered by our data, the end state for productive refolding of I27–I28 is FU/UF (see main text); rate coefficients in the figure are for I27–I27. ‘*E*' is the mean transfer efficiency determined in the global fit of the kinetic model; colour code as in Figs 3 and 4.

Data sets in Fig. 4 are fitted to a binding model describing the effective interaction of the polypeptide chain with GdmCl, according to^51^:


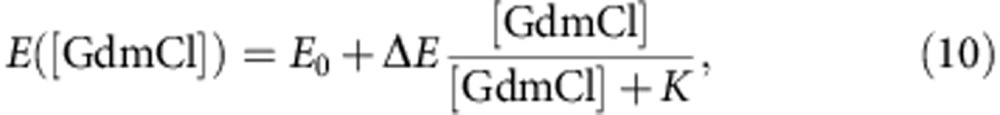


where *E* is the measured mean transfer efficiency, *E*_0_ is the transfer efficiency of the polypeptide chain in the absence of GdmCl, Δ*E*=*E*_0_−*E*_∞_ is the change in transfer efficiency on saturation of the chain with GdmCl, [GdmCl] is the concentration of GdmCl and *K* is the effective dissociation constant.

### Simulation methods

The same coarse-grained model for titin I27–I27 was used here as in previous work^27^. That is, a coarse-grained Gō-like model was generated based on the structure of I27 (1tit.pdb^52^) using a standard procedure^53^, with one bead per residue centred on the alpha carbon. Two identical I27 sequences were linked by four-residue repulsive linkers to create a model for two-domain tandems. Interactions between residues *i* and *j* in different domains were treated exactly like the interactions between those residues in the same domain, and interactions with the linker were repulsive. In addition to the native-like interactions, additional favourable self-interactions between each pair of residues with indices *i*,*i*+93 were added for selected residues. Those were the residues having a contact energy below zero in the PASTA^34^ knowledge-based potential (other residues retained the default repulsive potential from the Gō-like model). These residues interacted via the same pair potential as in the original model^17^,^53^, with a contact distance *σ*(*i,j*)=0.6 nm and contact energy *ɛ*(*i,j*)=*ɛ*_PASTA_(*i,j*)+Δ*ɛ* for residues *i* and *j*, where *ɛ*_PASTA_(*i,j*) is the relevant entry in the PASTA matrix for parallel β-interactions and Δ*ɛ* is an empirical offset (needed for any statistical potential). A value of –2.75 *k*_B_*T* for the offset was found to give a maximum transient population of the M2 intermediate most consistent with experiment. Folding simulations were run, starting from fully extended configurations, using Langevin dynamics with a friction of 0.1 ps^−1^ and a time step of 10 fs. A total of 128 independent runs initiated with different random seeds were performed, each for a duration of 10 μs. A modified version of the GROMACS 4.0.5 molecular simulation package was used for all calculations^54^. All the protein structures were rendered with the program PyMOL (Schrödinger).

### Assignment of states in trajectories

The states shown in Fig. 3e were determined via a transition-based assignment using contacts. A set of native contact pairs *S*_native_={(*i*_1_,*j*_1_), (*i*_2_,*j*_2_), …, (*i*_M_,*j*_M_)} was determined based on the native structure using the standard procedure for generating the Gō-type model^17^,^53^. Then native-like contact sets for ‘central domains' and ‘terminal domains' were generated as *S*_CD,*k*_={(*i*_1_+*θ*(*k*−*i*_1_)*L*, *j*_1_+*θ*(*k*−*j*_1_)*L*), (*i*_2_+*θ*(*k*−*i*_2_)*L*, *j*_2_+*θ*(*k*−*j*_2_)*L*) …} and *S*_TD,*k*_={(*i*_1_+*θ*(*i*_1_−*k*)*L*, *j*_1_+*θ*(*j*_1_−*k*)*L*), (*i*_2_+*θ*(*i*_2_−*k*)*L*, *j*_2_+*θ*(*j*_2_−*k*)*L*) …}, where *θ*(*x*) is the Heaviside step function and *L=*93 is the length of a titin domain and linker. The index *k* is needed to distinguish the different possible native-like misfolds. It corresponds to the point in the sequence where a loop is cut to make the central domain misfold and takes values *k*∈*K*={0, 16, 28, 37, 53, 64, 76}. For a native fold, *k*=0 and *S*_CD,0_ and *S*_TD,0_ are the sets of contacts for the N terminal and C terminal correctly folded domains. In addition, a set of non-native-like contacts *S*_PASTA_={(*i*, *i+L*) : *ɛ*_PASTA_(*i*,*i*)<0} was determined. For each set of contacts *S*_α_, a corresponding fraction of contacts could be determined as *Q*_α_ using:


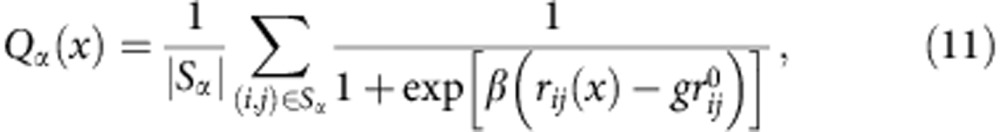


where *r*_*ij*_(*x*) is the distance between beads (residues) *i* and *j* in configuration *x*, and 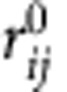 is the corresponding distance in the native structure for native-like contacts or 0.6 nm for non-native-like contacts; the factor *g*=1.2 allows for thermal fluctuations in the distance between residues which are in contact.

The system was initialized in the unfolded state, and then made transitions to another state only if it reached the ‘core region' for that state, defined as:

‘UU': configurations with *Q*_CD,0_<0.4, Q_TD,0_<0.4, *Q*_MU_<0.4, *Q*_UM_<0.4 and *Q*_PASTA_<0.4.

‘MU': configurations with *Q*_MU_>0.8 and *Q*_UM_<0.5

‘UM': configurations with *Q*_UM_>0.8 and *Q*_MU_<0.5

‘M3': configurations with *Q*_MM_>0.8

‘FU': configurations with *Q*_CD,0_>0.9 and *Q*_TD,0_<0.5

‘UF': configurations with *Q*_TD,0_>0.9 and *Q*_CD,0_<0.5

‘FF': configurations with (*Q*_CD,0_+*Q*_TD,0_)/2>0.9

‘M2': configurations with *Q*_PASTA_>0.8

For the above, we define *Q*_MU_=max_*k*≠0_{*Q*_CD,*k*_}, *Q*_UM_=max_*k*≠0_{*Q*_TD,*k*_} and *Q*_MM_=max_*k*≠0_{(*Q*_CD,*k*_+*Q*_CD,*k*_)/2}.

The lumped states ‘M1' and ‘FU/UF' are defined as: M1=UM ∪ MU and UF/FU=UF ∪ FU.

### Simulation models for central domain misfolds

Models for the circular permutants of I27 representing the central domains were constructed by simulating a chain consisting of residues *k*+1 to *k*+93 from the full I27–I27 sequence, where *k* is taken from the set *K* above. The energy function was otherwise identical to that used in the original Gō-like simulations^27^. Folding free energy surfaces *F*(*Q*) for the relevant fraction of native-like contacts (that is, *Q*_CD,k_) were determined for each circular permutant using umbrella sampling on that coordinate, combining the data with weighted histogram analysis^55^. The stability Δ*G*_fold_ was obtained as:


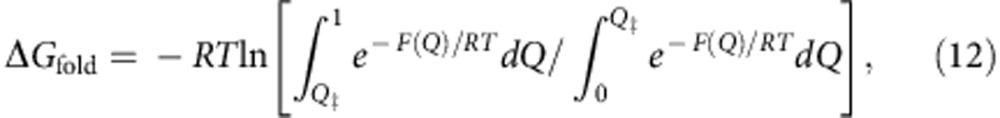


where *R* is the molar gas constant, *T* is the temperature and the position of the dividing surface between folded and unfolded is taken as *Q*_‡_=0.4. The free energy surfaces and stabilities are summarized in Supplementary Fig 5b.

## Additional information

**How to cite this article:** Borgia, A. *et al.* Transient misfolding dominates multidomain protein folding. *Nat. Commun.* 6:8861 doi: 10.1038/ncomms9861 (2015).

## Supplementary Material

Supplementary Information Supplementary Figures 1-9, Supplementary Table 1, Supplementary Discussion and Supplementary References
