# Supplementary material for: Transient misfolding dominates multidomain protein folding
Source: Nat Commun. 2015 Nov 17;6:8861. doi: 10.1038/ncomms9861 (PMC4660218; doi:10.1038/ncomms9861)
Supplement: Supplementary Information — Supplementary Figures 1-9, Supplementary Table 1, Supplementary Discussion and Supplementary References [file ncomms9861-s1.pdf]

Supplementary Figures

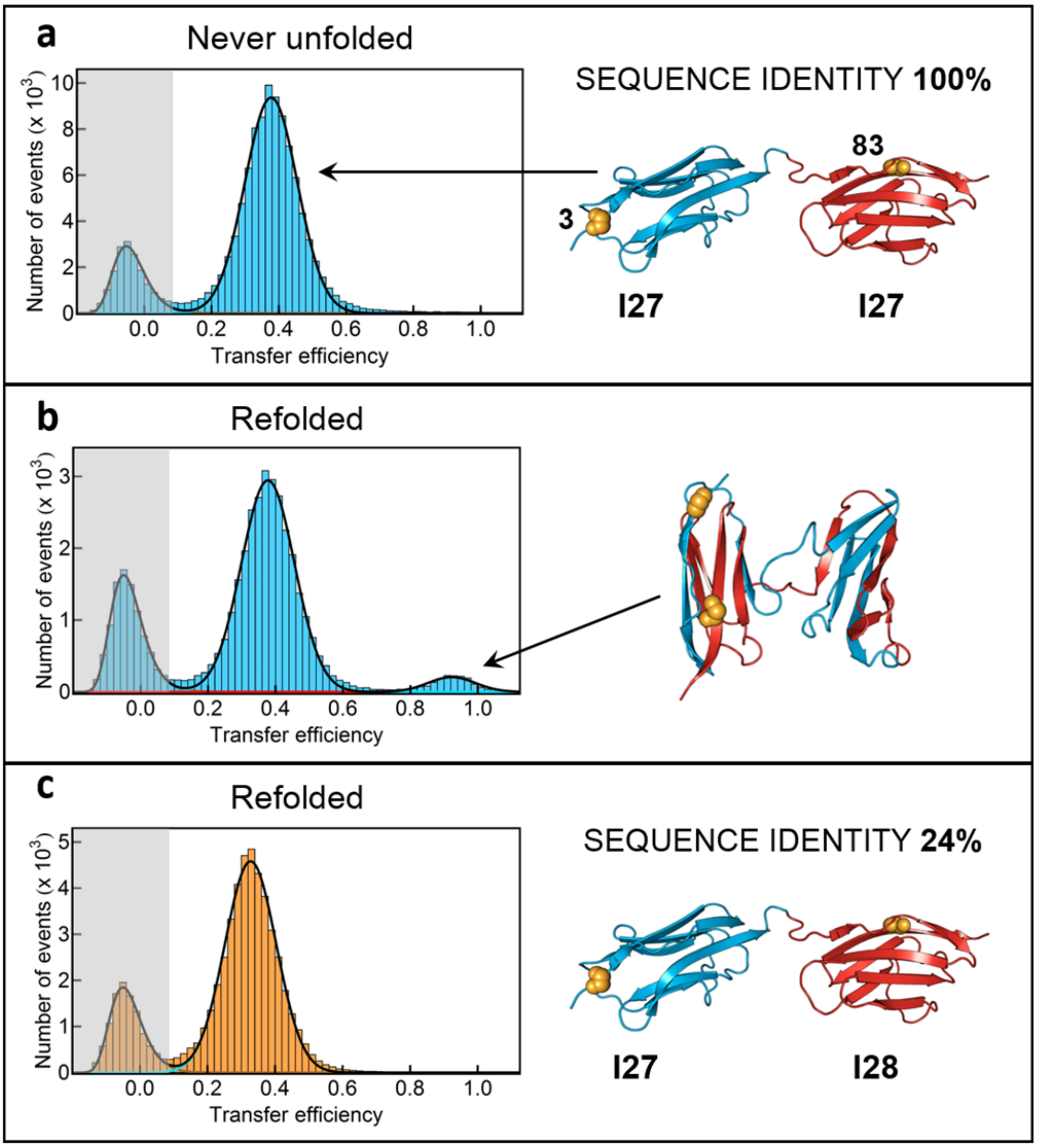

Supplementary Figure 1. Overview of refolding and misfolding for I27–I27 and I27–I28 tandem repeats observed in previous single-molecule FRET experiments.[1] a. Transfer efficiency histogram of never-unfolded I27–I27, with only the native state present at $E \approx 0.38$, shown alongside a representative structure of the protein, with the residues labelled with fluorescent dyes indicated as orange spheres. **b.** Histogram of I27–I27 after refolding by dilution from high GdmCl concentration, showing the formation of a long-lived misfolded population at $E \approx 0.9$, displayed with a representative structure of this species based on Gō-like simulations. **c.** Histogram of I27–I28 after refolding: the low sequence identity between the two naturally neighbouring domains prevents formation of the stable misfolded states seen in b. The structure shown is based on I27-I27. The area shaded in gray indicates the population of molecules lacking an active acceptor fluorophore (“donor-only”). Data taken from ref.[1]

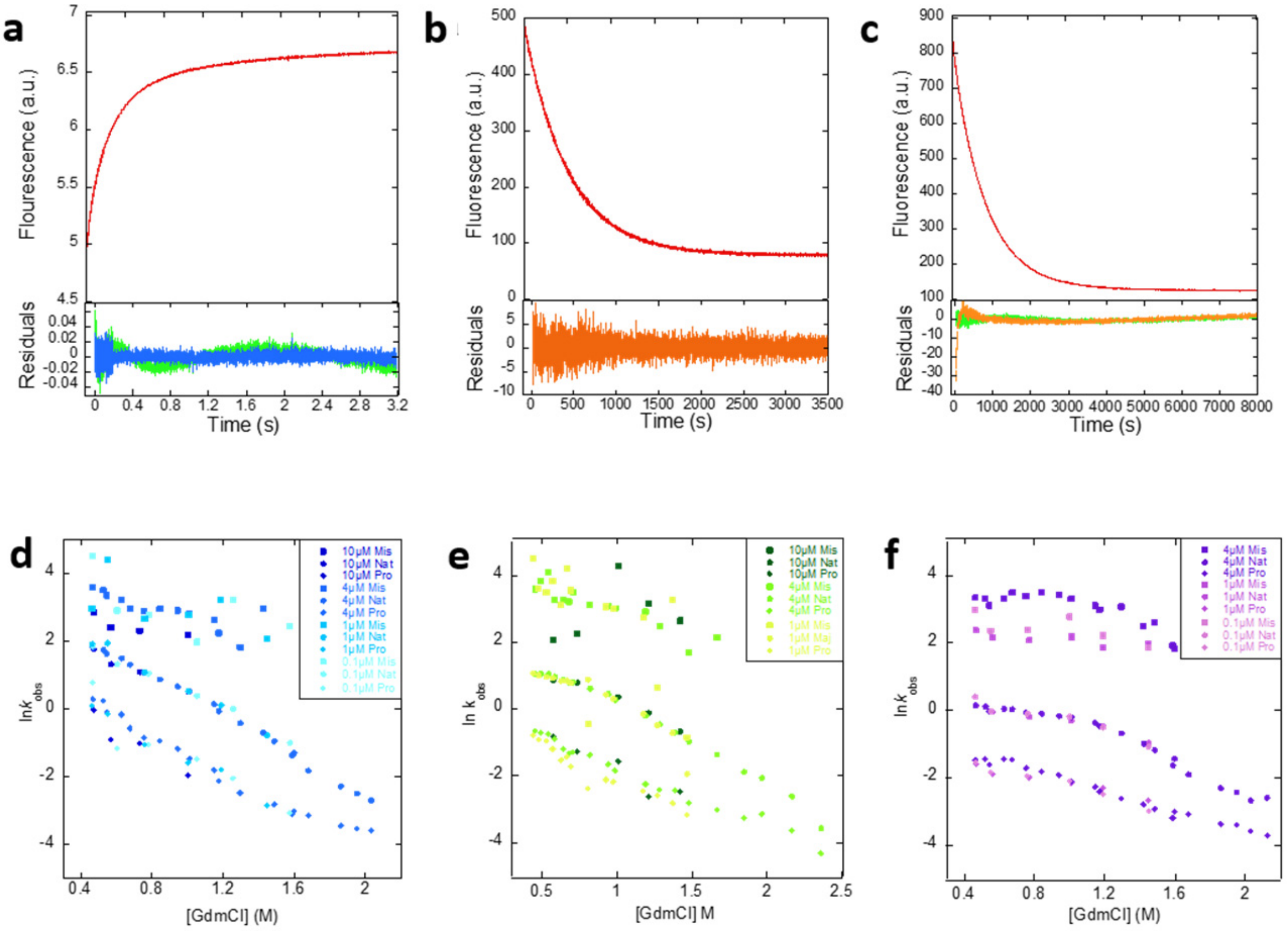

Supplementary Figure 2. Representative stopped-flow fluorescence kinetics traces and concentration dependence of refolding for I27 tandem repeats. a. Refolding kinetics. **b and c.** Unfolding kinetics of (b) “never unfolded” and (c) previously refolded protein. A second faster phase is observed in the previously refolded sample. In the tandem proteins, three refolding phases are observed, which we assign to native folding (Nat), misfolding (Mis) and peptidyl-prolyl cis-trans isomerization (Pro). Residuals for single-exponential fits are shown in orange, for double-exponential fits in green, and for triple-exponential fits in blue. **d-f.** Logarithm of observed relaxation rate coefficients (k_{obs} in s^{-1}) from experiments repeated at a range of final protein concentrations for (d**)** 2-domain, (e) 3-domain and (f) 8-domain I27 repeat constructs. The extent of rollover observed for the native phase (circles) is not affected by the protein concentration, indicating the absence of intermolecular aggregation.

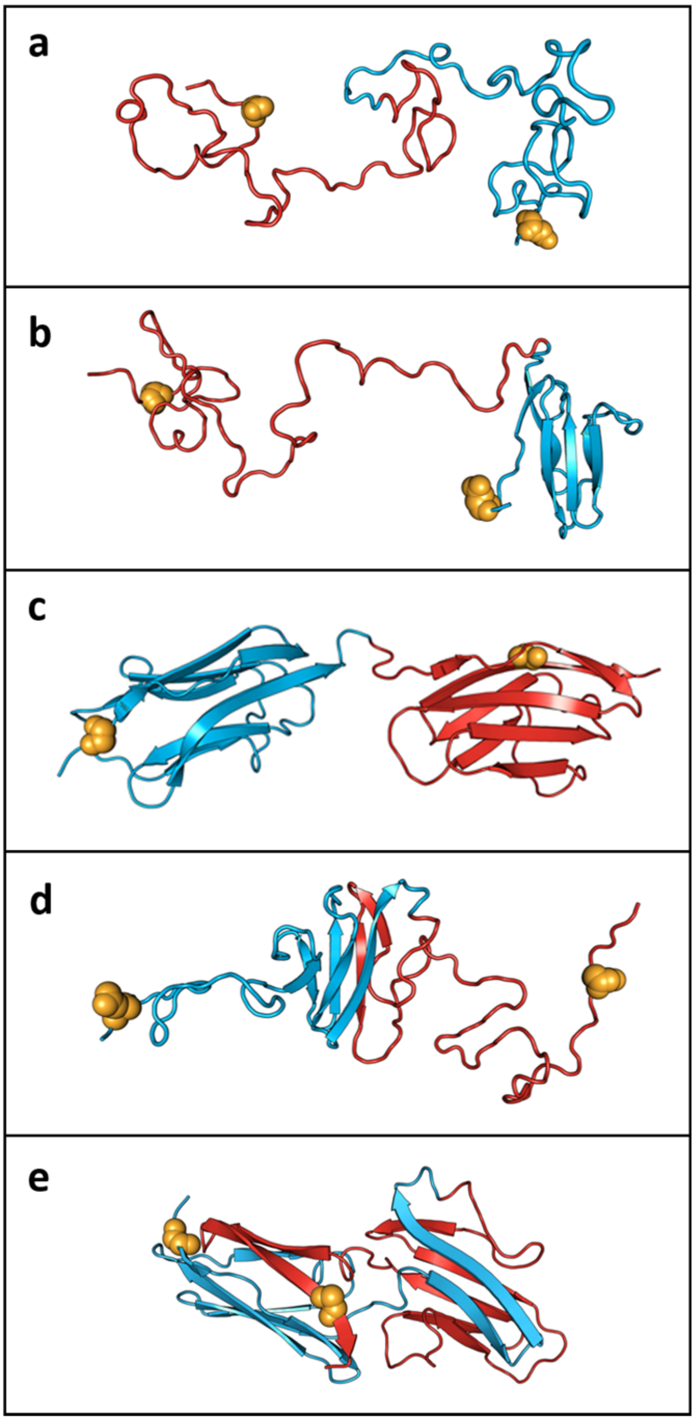

Supplementary Figure 3. Structural representations of the various species predicted to be populated during the refolding of I27–I27 (from Gō-type simulations). Representative structures of (a) the unfolded state, (b) the state with one domain correctly folded and the other unfolded, (c) the native state, (d) a strand-swapped misfolded state with the "central domain" folded and the "terminal domain" unfolded (see Results section), (e) the stable misfolded species. The residues labelled with fluorescent dyes are indicated as orange spheres.

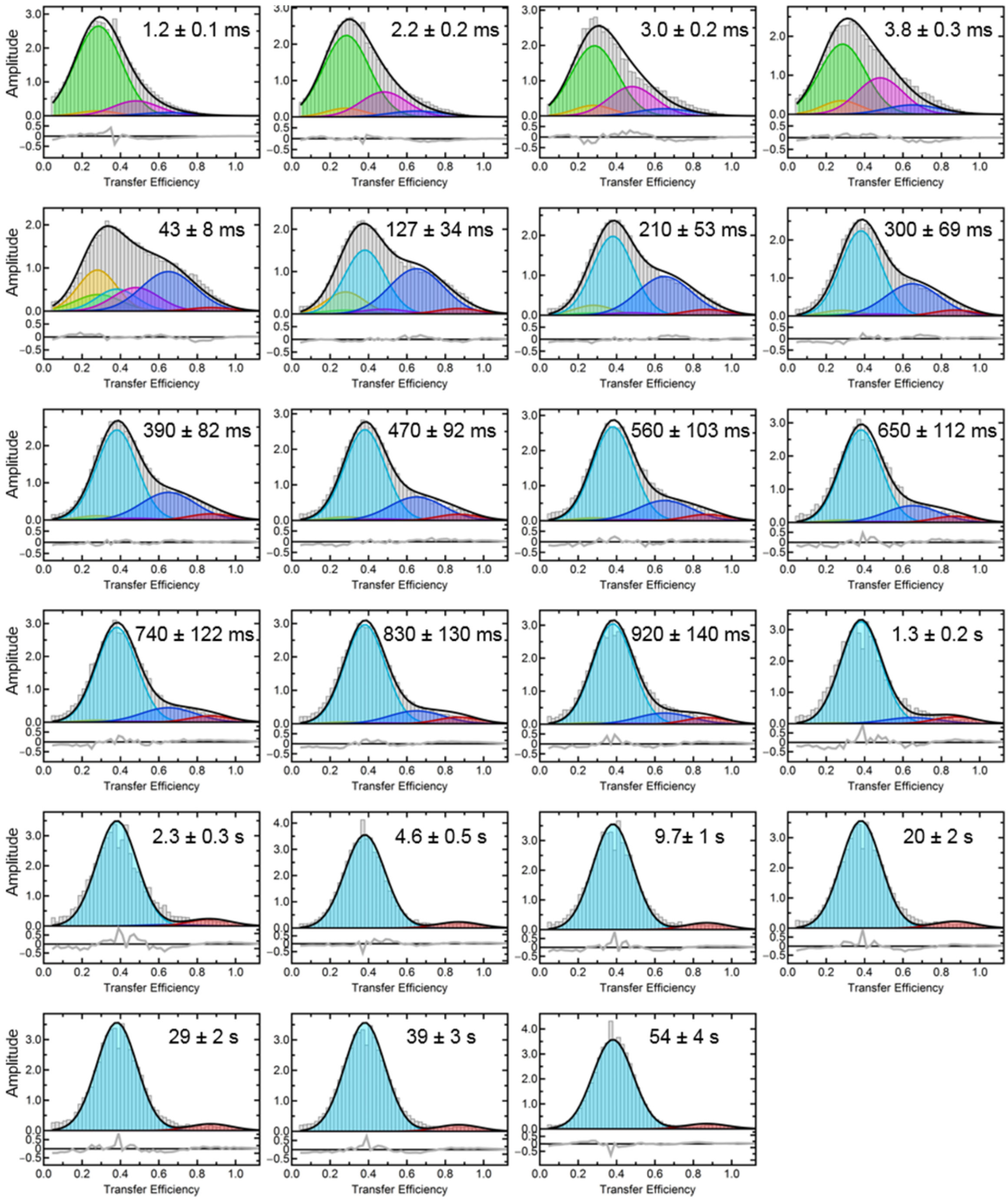

Supplementary Figure 4. The whole time series of FRET efficiency histograms for I27–I27 refolding. FRET efficiency histograms recorded during refolding of doubly-labelled I27–I27 after GdmCl dilution from 4.56 to 0.23 M (see Methods; time from mixing and corresponding uncertainty is given in the panels), ranging from 1.2 ± 0.1 ms to 54 ± 4 s. Fits of Gaussian peak functions corresponding to individual populations are color-coded as in Fig. 3; the respective sums of all Gaussians are shown as black lines. The gray lines in the panels below each histogram show the residuals calculated per bin, according to Eq. 9 (Methods). Every histogram shown is the average of two or more independent measurements.

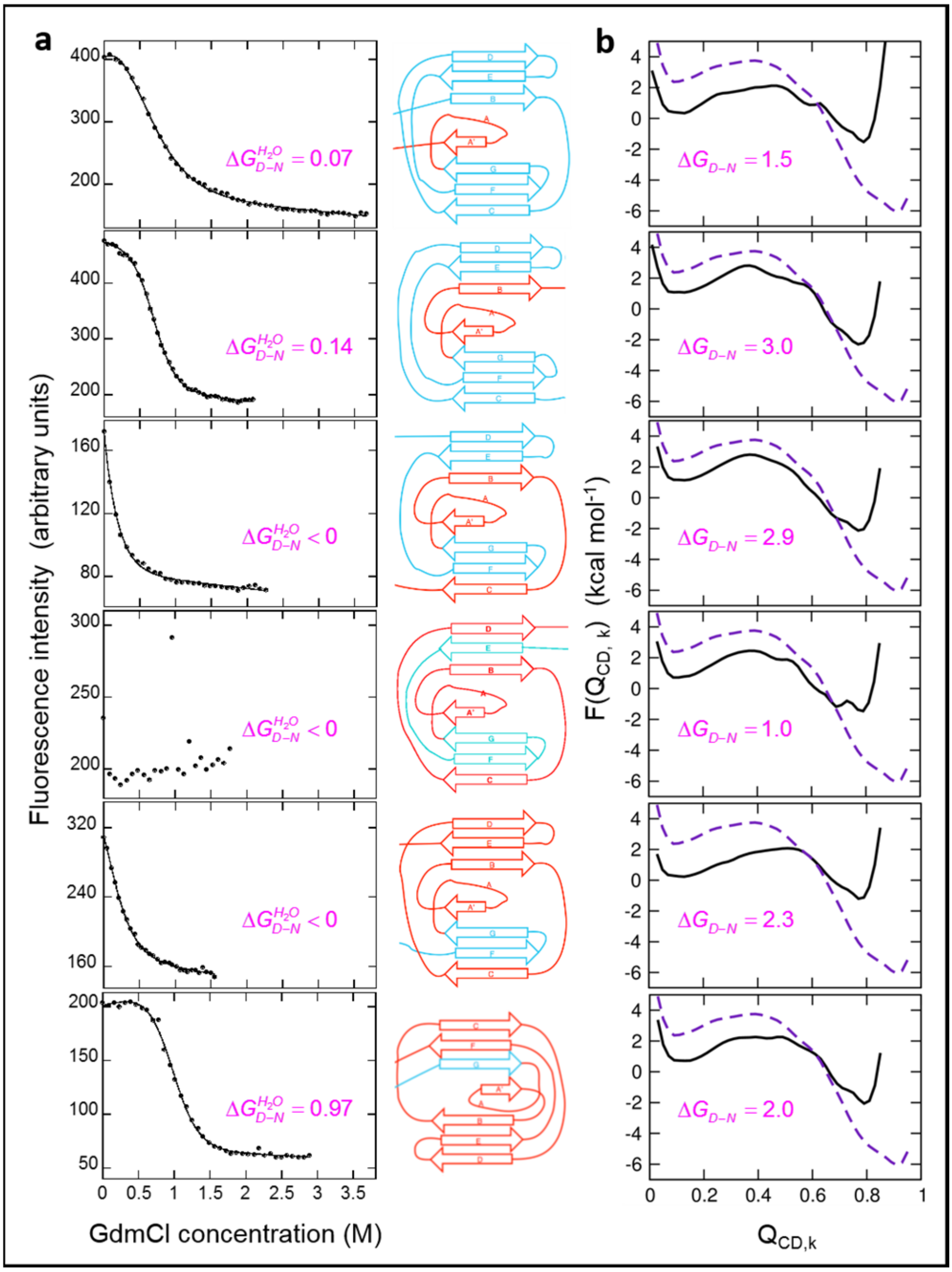

Supplementary Figure 5. Thermodynamic stabilities of I27 circular permutants from experiments and simulations. a. Equilibrium denaturation curves of the circular permutants in the presence of 1 M glucose. Denaturation free energies in water ($\Delta G_{D-N}^{H_2O}$) were calculated by subtracting the stabilisation from glucose, as explained in Methods. **b.** For each circular permutant we show the free energy surface F(Q) and the stability (ΔG_{D-N}) obtained from umbrella sampling simulations (Eq. 12), as explained in Methods. Topological diagrams of each circular permutant are shown in the centre. Free energies are in in kcal/mol.

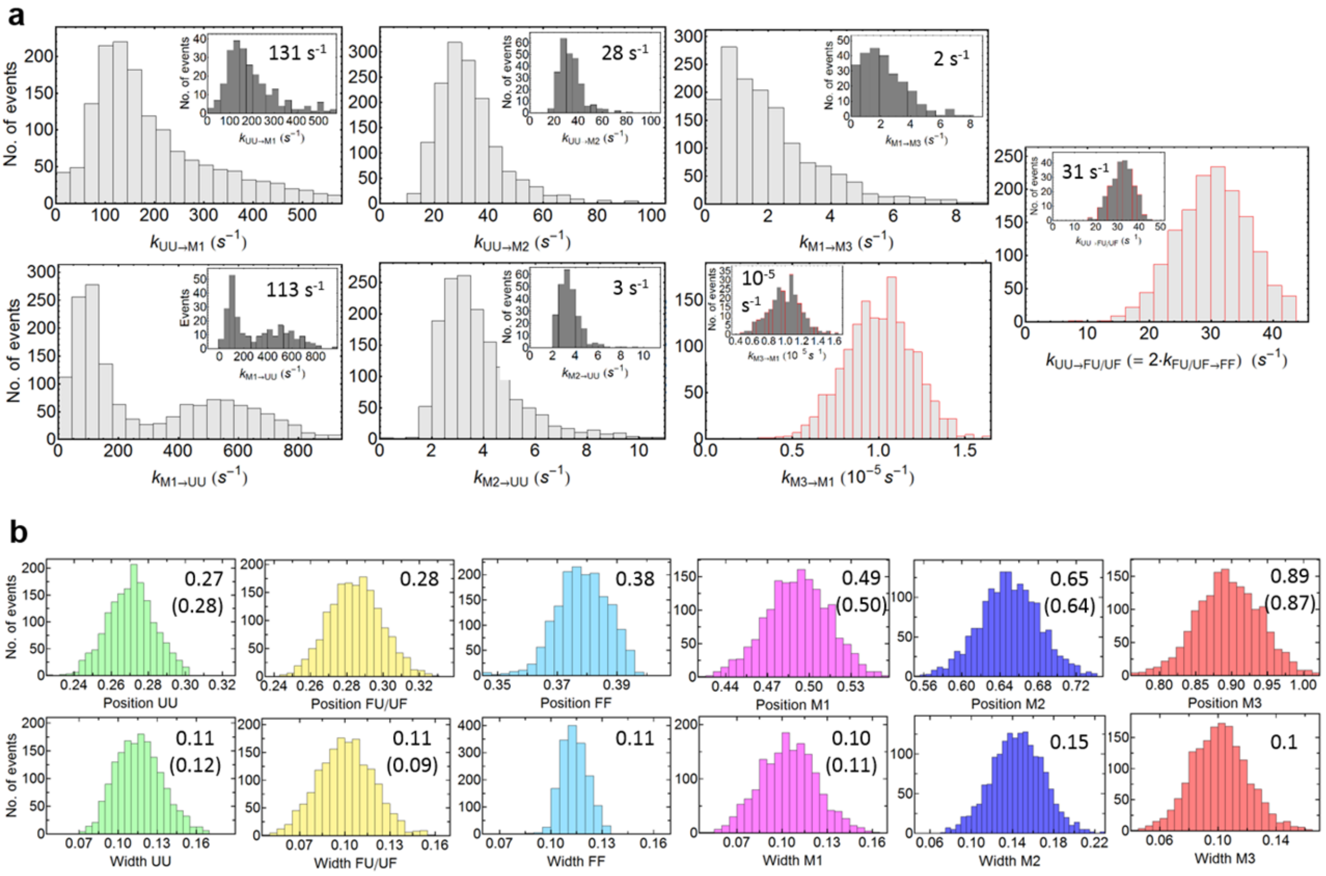

Supplementary Figure 6. Results of fit robustness test. a. Histograms of direct and inverse rate coefficients obtained from fits of the time-dependent transfer efficiency histograms (depicted in Supplementary Fig. 4), using 10^4 cycles of randomization of the fixed fit parameters (see Methods and Supplementary Table 1). Rate coefficients resulting from fits yielding a χ^2 within 50% (main histograms, light grey) or 5% (insets, dark grey) of the best χ^2, are shown, together with the value of the distribution maximum for the 5% threshold; histograms with bars lined in red indicate rate coefficients fixed and randomized together with all peak positions and widths prior to the procedure. **b**. Histograms of positions and widths for every population yielding the rate coefficient distribution in a (light grey). The values of the distribution maxima for χ^2 within 50% of the best χ^2 are provided, alongside analogous values for a χ^2 threshold of 5% when these are different (in brackets).

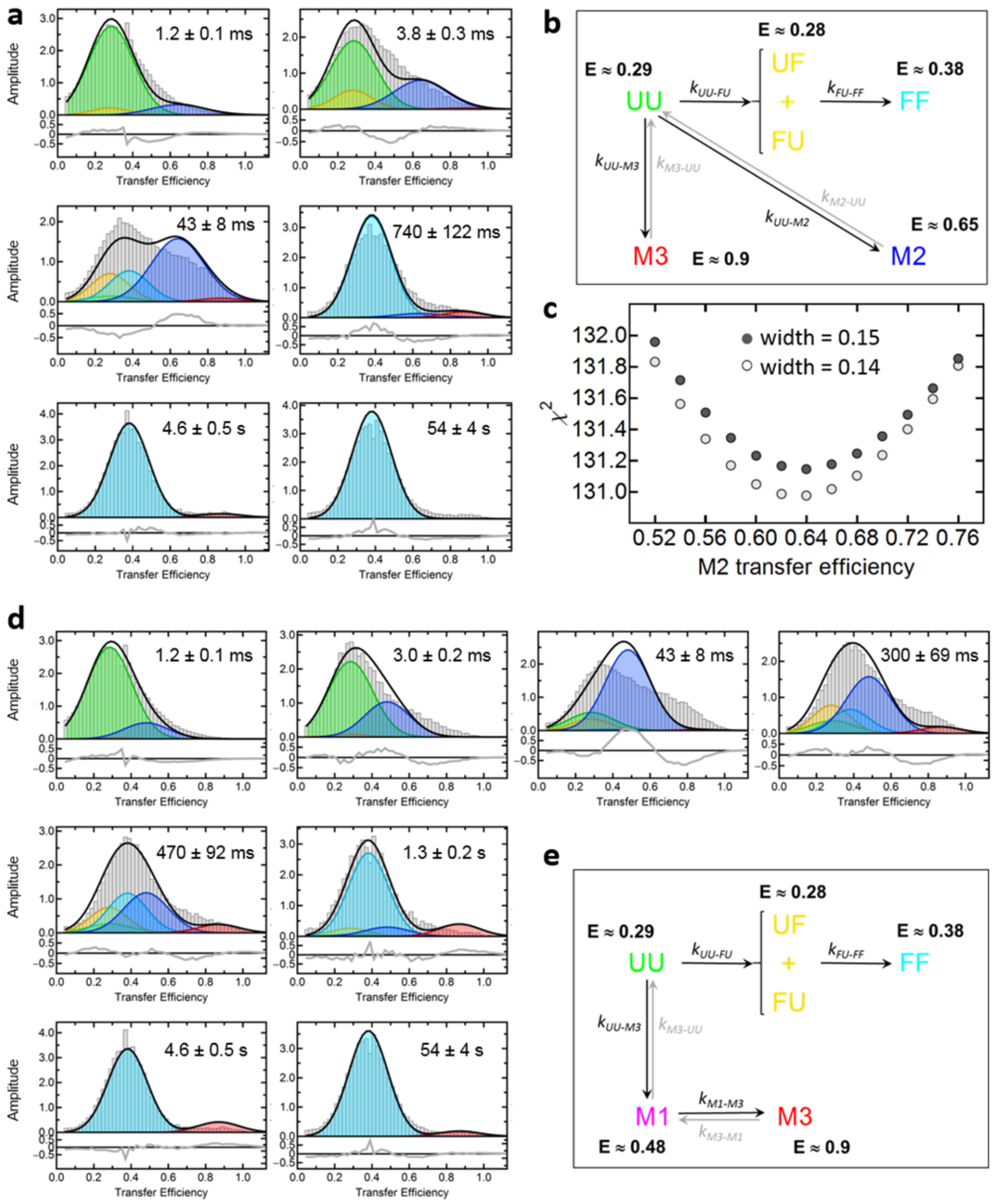

Supplementary Figure 7. Alternative fits of the whole time series of FRET efficiency histograms for I27–I27 refolding. A selection of FRET efficiency histograms of I27–I27 refolding, fitted to 5-species models (instead of 6) excluding either the M1 (**a**) or the M2 (**d**) population. Fits of Gaussian peak functions corresponding to individual populations are color-coded as in Fig. 3; the respective sums of all Gaussians are shown as black lines. The gray lines in the panels below each histogram show the residuals calculated per bin, according to Eq. 9 (Methods). The lower quality of these fits compared to the 6-species depicted in Supplementary Fig. 4 are evident from the lack of agreement between the fitted populations and the experimental data (quantified by the residuals below the panels). **b.** Kinetic scheme for the 5-state fit in a. **c.** A plot of the fit's χ^2 as a function of M2's transfer efficiency for two different peak widths; χ^2 for the 6-species fits is about 22 (see Methods and Supplementary Table 1). **e**. Kinetic scheme for the 5-state fit in d.

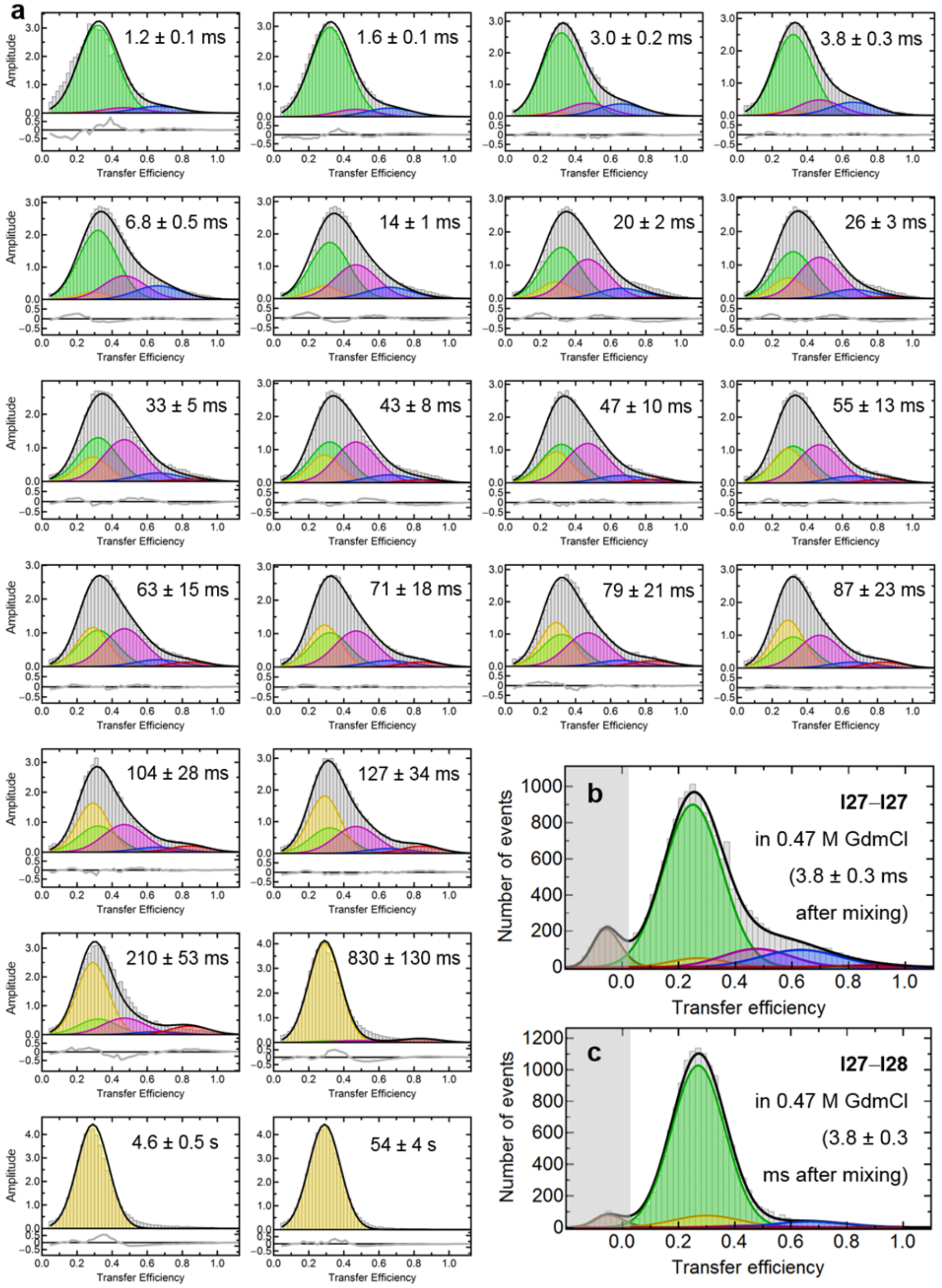

Supplementary Figure 8. **Time series of FRET efficiency histograms for I27–I28 refolding and comparison with I27–I27 at higher GdmCl concentration. a.** FRET efficiency histograms recorded during refolding of doubly-labelled I27–I28 after GdmCl dilution from 4.56 to 0.23 M (see Methods; time from mixing and relative uncertainty is given in the panels), ranging from 1.2 ± 0.1 ms to 54 ± 4 s. Fits of Gaussian peak functions corresponding to individual populations are color-coded as in Fig. 2 and 3; the respective sums of all Gaussians are shown as black lines. The gray lines in the panel below each histogram show the residuals calculated per bin, according to Eq. 9 (Methods). Every histogram shown is the average of two or more independent measurements. **b, c**. Transfer efficiency histograms of the refolding of doubly-labelled (b) I27–I27 and (c) I27–I28 in the microfluidic mixer 3.8 ± 0.3 ms after mixing, at ~0.47 M GdmCl, the lowest concentration used in ensemble refolding experiments (vs. 0.23 M, as shown in Fig. 3 and Supplementary Fig. 4 and 8a). Fits of Gaussian peak functions corresponding to individual populations are color-coded as in Fig. 3, and the sums of all curves are shown as a black lines; the area shaded in gray indicates the population of molecules lacking an active acceptor fluorophore ("donor-only" population). Although all misfolded populations are still present in (a), they are greatly reduced in (b).

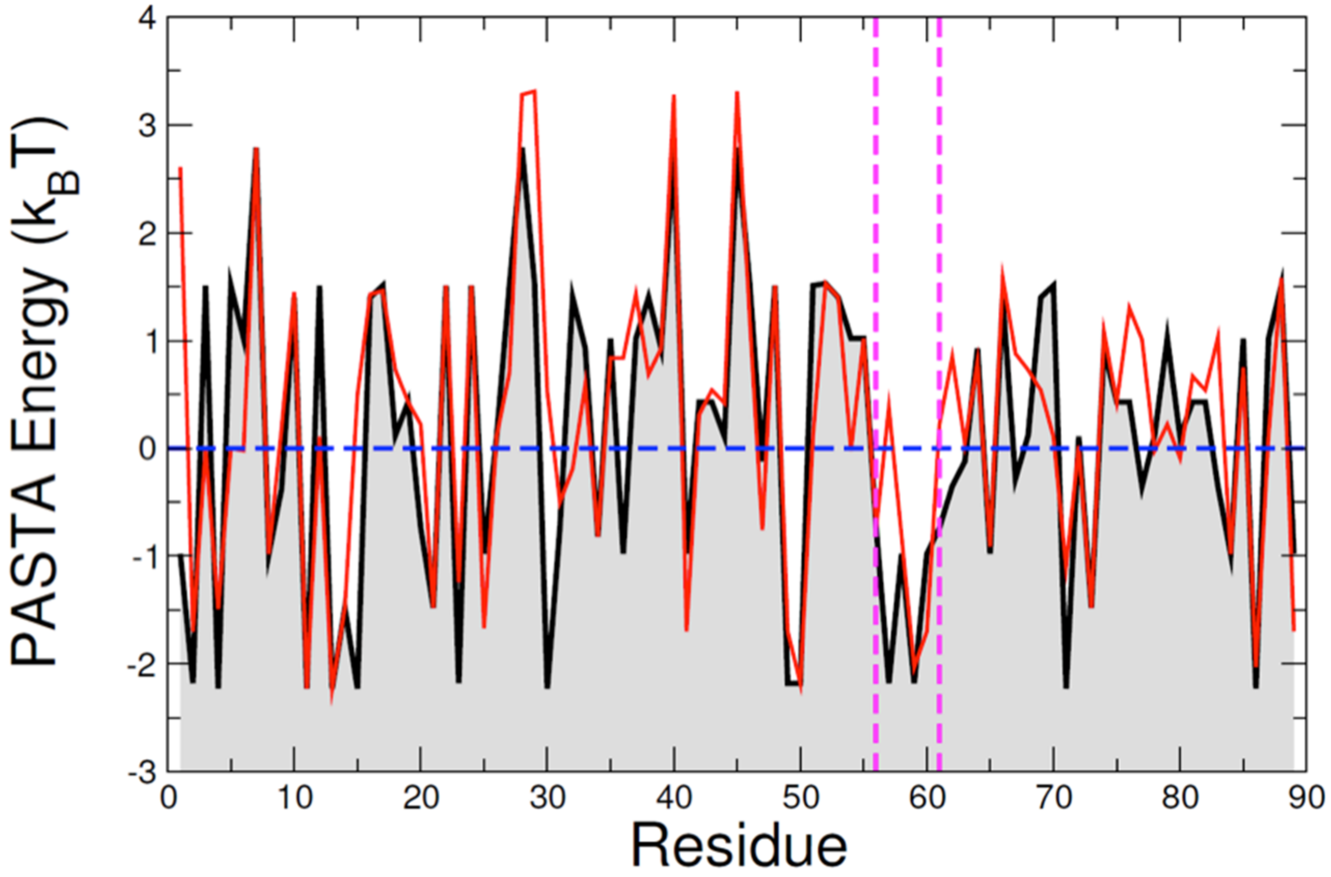

Supplementary Figure 9. Propensity for parallel beta-sheet formation in I27-I27 and I27-I28. Energies for *i,i+93* interactions in two-domain constructs, taken from the PASTA potential for parallel β-sheets (see Methods). Black curve gives energies for I27–I27, red curve for I27–I28. Vertical magenta lines indicate the region previously identified as most amyloid-prone[2]. Correspondingly, the regions with the lowest energies are most frequently involved in forming misfolded structures in the transiently formed M2 population (Fig. 4, Supplementary Fig. 2d).

I27–I27

	UU ⟨E⟩	UU Width	FU/UF ⟨E⟩	FU/UF Width	FF ⟨E⟩	FF Width	M1 ⟨E⟩	M1 Width	M2 ⟨E⟩	M2 Width	M3 ⟨E⟩	M3 Width
Best fit parameters	0.285	0.12	0.28	0.10	0.38	0.11	0.48	0.12	0.65	0.14	0.87	0.11
Starting point for automatic parameters randomization	(± 0.02)[a] 0.27	(± 0.01)[b] 0.11	(± 0.02)[a] 0.28	(± 0.01)[b] 0.10	(± 0.02)[c] 0.38	(± 0.01)[b] 0.10	(± 0.03)[a] 0.49	(± 0.01)[b] 0.10	(± 0.02)[a] 0.65	(± 0.01)[b] 0.14	(± 0.03)[c] 0.90	(± 0.01)[b] 0.10
Distribution maxima for 10^4 iterations of randomization ($\chi^2 \leq 1.05\ \chi^2_{best}$)	(± 0.01)[d] 0.28	(± 0.01)[d] 0.11	(± 0.01)[d] 0.28	(± 0.02)[d] 0.10	($\pm 5\cdot10^{-3}$)[d] 0.38	($\pm 4\cdot10^{-3}$)[d] 0.11	(± 0.02)[d] 0.49	(± 0.02)[d] 0.11	(± 0.03)[d] 0.64	(± 0.02)[d] 0.15	(± 0.04)[d] 0.87	(± 0.02)[d] 0.10

	$k_{UU\to FU}$	$k_{FU/UF\to FF}$	$k_{UU\to M1}$	$k_{UU\to M2}$	$k_{M1\to UU}$	$k_{M2\to UU}$	$k_{M1\to M3}$	$k_{M3\to M1}$
Best fit parameters	32	16	129	31	113	3	2	$2\cdot10^{-3}$
Starting point for automatic parameters randomization	30 (random.)	15 (random.)	176 (fitted)	28 (fitted)	227 (fitted)	2 (fitted)	2 (fitted)	10^{-5} (random.)
Distribution maxima for 10^4 iterations of randomization ($\chi^2 \leq 1.05\ \chi^2_{best}$)	31 ± 5 [d]	$k_{UU\to FU}$ / 2	131 ± 63 [d]	28 ± 10 [d]	113 ± 52 [d]	3 ± 1 [d]	2 ± 1 [d]	$2\cdot10^{-5} \pm 10^{-6}$ [d]

I27–I28

	UU ⟨E⟩	UU Width	FU/UF ⟨E⟩	FU/UF Width	FF ⟨E⟩	FF Width	M1 ⟨E⟩	M1 Width	M2 ⟨E⟩	M2 Width	M3 ⟨E⟩	M3 Width
Best fit parameters	0.32	0.11	0.29	0.09	-----	-----	0.47	0.12	0.66	0.13	0.87	0.11

	$k_{UU\to FU}$	$k_{FU/UF\to FF}$	$k_{UU\to M1}$	$k_{UU\to M2}$	$k_{M1\to UU}$	$k_{M2\to UU}$	$k_{M1\to M3}$	$k_{M3\to M1}$
Best fit parameters	10	-----	53	88	50	399	2	3

Supplementary Table 1. **Parameters for the global fit of 2-domain tandem repeats**. **a**. Uncertainty of the transfer efficiency values obtained from the extrapolation procedure described in main text "Results" is given by the 90% confidence interval at 0.23 M GdmCl (Fig. 4). ***b.*** Uncertainty of widths is the difference between the widths of FRET efficiency peaks constructed implementing a minimum threshold of 35 and 50 photons per burst (see Methods). ***c.*** Uncertainty of all the known transfer efficiency values is the expected variability of this parameter when measured on different instruments. ***d.*** Uncertainty is one standard deviation of the corresponding parameter distribution. Positions (⟨E⟩) and widths for each FRET efficiency population used for the kinetic global fit of the data in Supplementary Figs 4 and 8 a are given alongside the resulting rate coefficients (in s^{-1}). 'Best-fit parameters' were obtained by manual variation within the uncertainty interval, aimed at minimizing χ^2. 'Starting point parameters' are the values obtained from independent measurements (except for M2) and randomized in our computational procedure to assess fit robustness; distributions of parameters values from which we obtained the "distribution maxima" are reported in Supplementary Fig. 7. Rate coefficients obtained using either manual optimization or random sampling of parameter space (see Methods) are virtually identical and robust to multiple simultaneous parameter variations, with only the rate coefficients for the UU ↔ M1 interconversion showing a noticeable variability for non-optimized parameters. Parameters are colour-coded according to the species as in all other figures.

Supplementary Discussion

Rationalizing Ensemble Folding Kinetics.

The fast phase in the ensemble folding kinetics arises in the kinetic model through parallel formation of the misfolded states M1 and M2, as well as direct folding to the native state (without first misfolding). Although each M1 misfold is likely to form more slowly than the native, there are at least five different strand-swapped topologies with native-like structure that contribute to the depletion of the unfolded state fluorescence in parallel pathways, increasing the rate. The slow phase arises mainly from correct folding to FF, after initial trapping in one of the misfolded states.

Ensemble refolding kinetics of I27–I28, 2- and 8-domain constructs.

In contrast to I27–I27[1], ensemble refolding and unfolding experiments for tandem I27–I28 2- and 8-domain constructs did not yield additional phases in either folding or unfolding experiments, nor any roll-over in the folding arm of the chevron indicative of sequestering of the protein in non-native species. However, the stopped-flow data can only be collected at higher GdmCl concentrations (≥0.45 M) than the single-molecule experiments (0.23 M). It is likely that the non-native species observed in I27–I28 are too unstable to be observed at higher denaturant concentrations. Indeed, in single-molecule experiments performed at 0.45 M, the same misfolded species as at 0.23 M are still detectable for I27–I27, but are negligible for I27–I28 (Supplementary Fig. 8 b and c).

Ensemble unfolding kinetics reveals increasing domain-swapped misfolding for multiple titin Ig-like repeats.

Considering that the stable misfolded state previously observed in single-molecule FRET experiments[1] (Fig. 1b and Supplementary Fig. 1b) is formed via the reciprocal swapping of β-strands between the two domains of the covalent tandem construct, the probability of forming such state should increase with the number of domains in the construct. This prediction, which was proven correct in single-molecule FRET experiments on a 3-domain tandem, can be tested in ensemble experiments

performing denaturant-dependent unfolding kinetics of constructs with 2, 3 or 8 tandem repeats of I27. Unfolding of newly expressed and purified tandem proteins ("never-unfolded" samples) resulted in a single-exponential fluorescence decay with rates identical, within experimental uncertainty, to those for the unfolding of an isolated I27 domain (Fig. 2a): we term this the "native unfolding phase". In contrast, proteins which had been unfolded in 5 M guanidinium chloride (GdmCl) for ≥ 2 hours, then refolded by dilution to a final concentration of 0.5 M GdmCl for 2 minutes, displayed unfolding kinetics better described by a double-exponential decay (Supplementary Fig. 2b-c). The rate coefficient for the major unfolding phase were the same for all tandem proteins and for their never-unfolded counterparts (Fig. 2a), indicating that this phase originates from unfolding of natively folded domains. The rate coefficients of the second, minor unfolding phase, however, were higher, but also invariant for all tandem proteins. Notably, the amplitude of this phase increased with the number of repeats in the tandem protein (Fig. 2c), suggesting that it originates from the unfolding of misfolded conformations. As This conclusion is supported by the agreement between the relaxation rate coefficients of the fast unfolding phase and the misfolded subpopulation unfolding measured in single-molecule FRET experiments (see "Ensemble kinetics and the effect of non-native interactions" in Results). For the 3- and 8-domain constructs, a simple calculation based solely on the number of covalently linked domains predicts that the proportion of misfolded domains would increase by a factor of 1.3 and 1.8, respectively, relative to the 2-domain tandem, in good agreement with the measured amplitude increase in Fig. 2b and 2c (1.4 ± 0.1 and 1.8 ± 0.1, respectively) (see Methods for details). These results suggest that domain-swapped misfolding can be an even greater problem for multidomain proteins with a large number of repeats.

Hypothesis for the formation of native-like, strand-swap central domain misfolds in I27-I28.

Studies of structurally related proteins have shown that folding rate coefficients and mechanism are strongly influenced by native state topology[3,4], and that both the transition state (TS) structure and folding mechanism tend to be conserved between members of the same family.[5,6] Several authors have highlighted that the structure of the swapped domains is also mainly determined by native topology[7-10]

and that most monomeric proteins in the same family or superfamily share a common swapped structure, which very often resembles closely the native monomer[10]. This suggests that topological and sequence determinants governing folding are likewise important for misfolding *via* domain swapping.

All Ig-like domains appear to fold *via* a nucleation-condensation mechanism, where the obligatory folding nucleus comprises a ring of highly conserved hydrophobic residues from each of the B, C, E and F-strands, interacting within the protein core and surrounded by a second order of conserved residues stabilizing this interaction network[11,12]. Early packing of these residues during folding establishes the correct Greek key topology of the native conformation, stabilizes the folding transition state, and ensures rapid end efficient folding[13]. Although I27 and I28 display a global sequence identity of 24%, the identity score rises to 40% (with an additional 33% of highly similar residues), if the comparison is limited to residues with ϕ-value $\geq$0.5, that is, those important for the stabilization of the folding TS.

This can explain why extensive formation of misfolded intermediates during I27–I28 refolding does not lead to a stable misfolded state at equilibrium. The $M^{27}_{CD} - U^{27}_{TD}$ misfolded states resemble folded domains and are thus likely to require the recruitment of the same conserved set of residues constituting the folding nucleus in order to form[11-14]. Such residues in I28 are probably similar enough to those of I27 to enable the formation of these native-like misfolded structures. However, the thermodynamic stability of such native-like misfolded states will still be determined by the size and strength of the whole interaction network, which depends on the overall sequence identity of the swapping partners, and that is probably too low for these species to be long-lived.

References

1 Borgia, M. B., Borgia, A., Best, R. B., Steward, A., Nettels, D. *et al.* Single-molecule fluorescence reveals sequence-specific misfolding in multidomain proteins. *Nature* **474**, 662-665 (2011).

2 Zheng, W. H., Schafer, N. P. & Wolynes, P. G. Frustration in the energy landscapes of multidomain protein misfolding. *Proc. Natl. Acad. Sci. USA* **110**, 1680-1685 (2013).

3 Ivankov, D. N., Garbuzynskiy, S. O., Alm, E., Plaxco, K. W., Baker, D. *et al.* Contact order revisited: Influence of protein size on the folding rate. *Protein Sci.* **12**, 2057-2062 (2003).

4 Plaxco, K. W., Simons, K. T. & Baker, D. Contact order, transition state placement and the refolding rates of single domain proteins. *J. Mol. Biol.* **277**, 985-994 (1998).

5 Gunasekaran, K., Eyles, S. J., Hagler, A. T. & Gierasch, L. M. Keeping it in the family: folding studies of related proteins. *Curr. Opin. Struct. Biol.* **11**, 83-93 (2001).

6 Zarrine-Afsar, A., Larson, S. M. & Davidson, A. R. The family feud: do proteins with similar structures fold via the same pathway? *Curr. Opin. Struct. Biol.* **15**, 42-49 (2005).

7 Ding, F., Prutzman, K. C., Campbell, S. L. & Dokholyan, N. V. Topological determinants of protein domain swapping. *Structure* **14**, 5-14 (2006).

8 Rousseau, F., Schymkowitz, J. W. & Itzhaki, L. S. The unfolding story of three-dimensional domain swapping. *Structure* **11**, 243-251 (2003).

9 Yang, S. C., Cho, S. S., Levy, Y., Cheung, M. S., Levine, H. *et al.* Domain swapping is a consequence of minimal frustration. *Proc. Natl. Acad. Sci. USA* **101**, 13786-13791 (2004).

10 Huang, Y., Cao, H. & Liu, Z. Three-dimensional domain swapping in the protein structure space. *Proteins* **80**, 1610-1619 (2012).

11 Bork, P., Holm, L. & Sander, C. The Immunoglobulin Fold - Structural Classification, Sequence Patterns and Common Core. *J. Mol. Biol.* **242**, 309-320 (1994).

12 Lappalainen, I., Hurley, M. G. & Clarke, J. Plasticity within the obligatory folding nucleus of an immunoglobulin-like domain. *J. Mol. Biol.* **375**, 547-559 (2008).

13 Geierhaas, C. D., Paci, E., Vendruscolo, M. & Clarke, J. Comparison of the transition states for folding of two Ig-like proteins from different superfamilies. *J. Mol. Biol.* **343**, 1111-1123 (2004).

14 Clarke, J., Cota, E., Fowler, S. B. & Hamill, S. J. Folding studies of immunoglobulin-like beta-sandwich proteins suggest that they share a common folding pathway. *Struct. Fold. Des.* **7**, 1145-1153 (1999).
